# Interplay between Side Chain Density and Polymer Alignment: Two Competing Strategies for Enhancing the Thermoelectric Performance of P3HT Analogues

**DOI:** 10.1021/acs.chemmater.3c01680

**Published:** 2023-10-26

**Authors:** Peter A. Gilhooly-Finn, Ian E. Jacobs, Olivier Bardagot, Yasser Zaffar, Antoine Lemaire, Shubhradip Guchait, Lu Zhang, Mark Freeley, William Neal, Fanny Richard, Matteo Palma, Natalie Banerji, Henning Sirringhaus, Martin Brinkmann, Christian B. Nielsen

**Affiliations:** † Department of Chemistry, University College London, Gower Street, London WC1E 6BT, U.K.; ‡ Department of Chemistry, Queen Mary University of London, Mile End Road, London E1 4NS, U.K.; § Optoelectronics Group, University of Cambridge, Cavendish Laboratory, J J Thomson Avenue, Cambridge CB3 0HE, U.K.; ∥ Department of Chemistry, Biochemistry and Pharmaceutical Sciences, University of Bern, Freiestrasse 3, 3012 Bern, Switzerland; ⊥ Charles Sadron Institute (ICS), CNRS Université de Strasbourg, UPR 22, 23 Rue du Loess, Strasbourg Cedex 02, 67034, France; # Université de Strasbourg, CNRS, ISIS UMR 7006, Strasbourg 67000, France

## Abstract

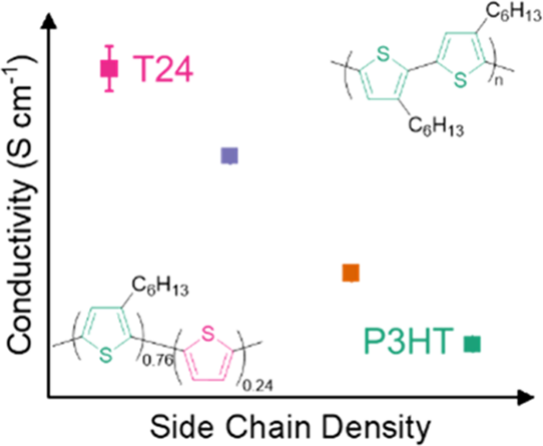

A series of polythiophenes with varying side chain density was synthesized, and their electrical and thermoelectric properties were investigated. Aligned and non-aligned thin films of the polymers were characterized in the neutral and chemically doped states. Optical and diffraction measurements revealed an overall lower order in the thin films with lower side chain density, also confirmed using polarized optical experiments on aligned thin films. However, upon doping the non-aligned films, a sixfold increase in electrical conductivity was observed for the polythiophene with the lowest side chain density compared to poly(3-hexylthiophene) (P3HT). We found that the improvement in conductivity was not due to a larger charge carrier density but an increase in charge carrier mobility after doping with 2,3,5,6-tetrafluoro-7,7,8,8-tetracyanoquinodimethane (F4TCNQ). On the other hand, doped aligned films did not show the same trend; lower side chain density instead led to a lower conductivity and Seebeck coefficient compared to those for P3HT. This was attributed to the poorer alignment of the polymer thin films with lower side chain density. The study demonstrates that optimizing side chain density is a synthetically simple and effective way to improve electrical conductivity in polythiophene films relevant to thermoelectric applications.

## Introduction

Organic semiconducting polymers are emerging as viable candidates for thermoelectric devices, in particular for low-grade heat harvesting and for applications where conformability is important.^1,2^ Side chains for organic semiconductors are crucial for solubilizing, otherwise insoluble, polymers, allowing device fabrication by solution processing.^3^ In addition to other advantages, side chains can also induce highly ordered solid-state morphologies needed for high charge transport.^4^ However, for organic semiconductors to be realized as a potential thermoelectric material, high charge carrier mobility needs to be coupled with high charge carrier density to improve their intrinsically low electrical conductivity.^5^

Increasing the charge carrier density can be achieved via doping, either chemically (also known as molecularly) or electrochemically, utilizing redox reactions that add an electron to the lowest unoccupied molecular orbital (LUMO) or remove an electron from the highest occupied molecular orbital (HOMO) for n-type or p-type doping, respectively. The chemical doping method is one of the most widely used techniques to improve the charge carrier density and therefore the electrical conductivity in an organic semiconducting polymer. For p-type doping, strong electron acceptors such as 2,3,5,6-tetrafluoro-7,7,8,8-tetracyanoquinodimethane (F4TCNQ), Mo(tfd-COCF_3_)_3_, and iron chloride (FeCl_3_) are frequently used in conjunction with electron-rich semiconducting polymers such as poly(3-hexylthiophene) (P3HT) and poly[2,5-bis(3-tetradecylthiophen-2-yl)thieno[3,2-*b*]thiophene] (PBTTT). On the other hand, n-type chemical doping is traditionally accomplished via a hydride source such as 4-(2,3-dihydro-1,3-dimethyl-1*H*-benzimidazol-2-yl)-*N*,*N*-dimethylbenzenamine (*N*-DMBI) and its derivates.^6^ Improvement in doping procedures has progressed from blending of the dopant and organic semiconductor to sequential doping techniques. Sequential doping techniques enable the highly ordered solid-state morphology to be retained to some extent due to the use of an orthogonal solvent. Utilizing the sequential doping method, Yamashita et al. reported the anion exchange doping method that allows for almost 100% doping efficiency, substantially improving the electrical conductivity.^7^

Besides creating extra charge carriers, structural changes to the semiconductor film also occur upon doping.^8^ Dopants can either co-crystallize with the semicrystalline polymer or disrupt the ordered packing, depending on the size and redox strength of the dopant, leading to a lower thermoelectric performance for the latter scenario. With this in mind, polymer–dopant pairs should be evaluated based not only on matching energetics for favorable electron transfer but also on steric considerations, such as dopant size, polymer side chain density, and side chain interdigitation, and non-covalent interactions between the polymer and the dopant.^9,10^

Much work has been carried out recently on polythiophenes, trying to understand where dopants reside in the solid state and how they may affect the electrical conductivity.^11−15^ Jacobs et al. recently showed that the overall crystalline structure of the film is the major driving force for high conductivity.^16^ They conducted a study on a range of semiconducting polymers, including P3HT and PBTTT, and showed that increasing the anion size when using ion exchange doping disrupted the packing to a greater extent, leading to lower conductivity. Doping of highly oriented semiconducting polymer films has also resulted in higher thermoelectric performance compared to non-aligned films. High-temperature rubbing on semicrystalline polymer films leads to higher conductivity and Seebeck coefficients in a direction parallel to the rubbing direction due to aligned crystalline domains affording efficient charge transport.^17^ However, high electrical conductivity in organic semiconductors usually requires a material design that produces highly crystalline films before doping or strategies to co-crystallize the dopant and the semiconductor.

**Figure 1:**
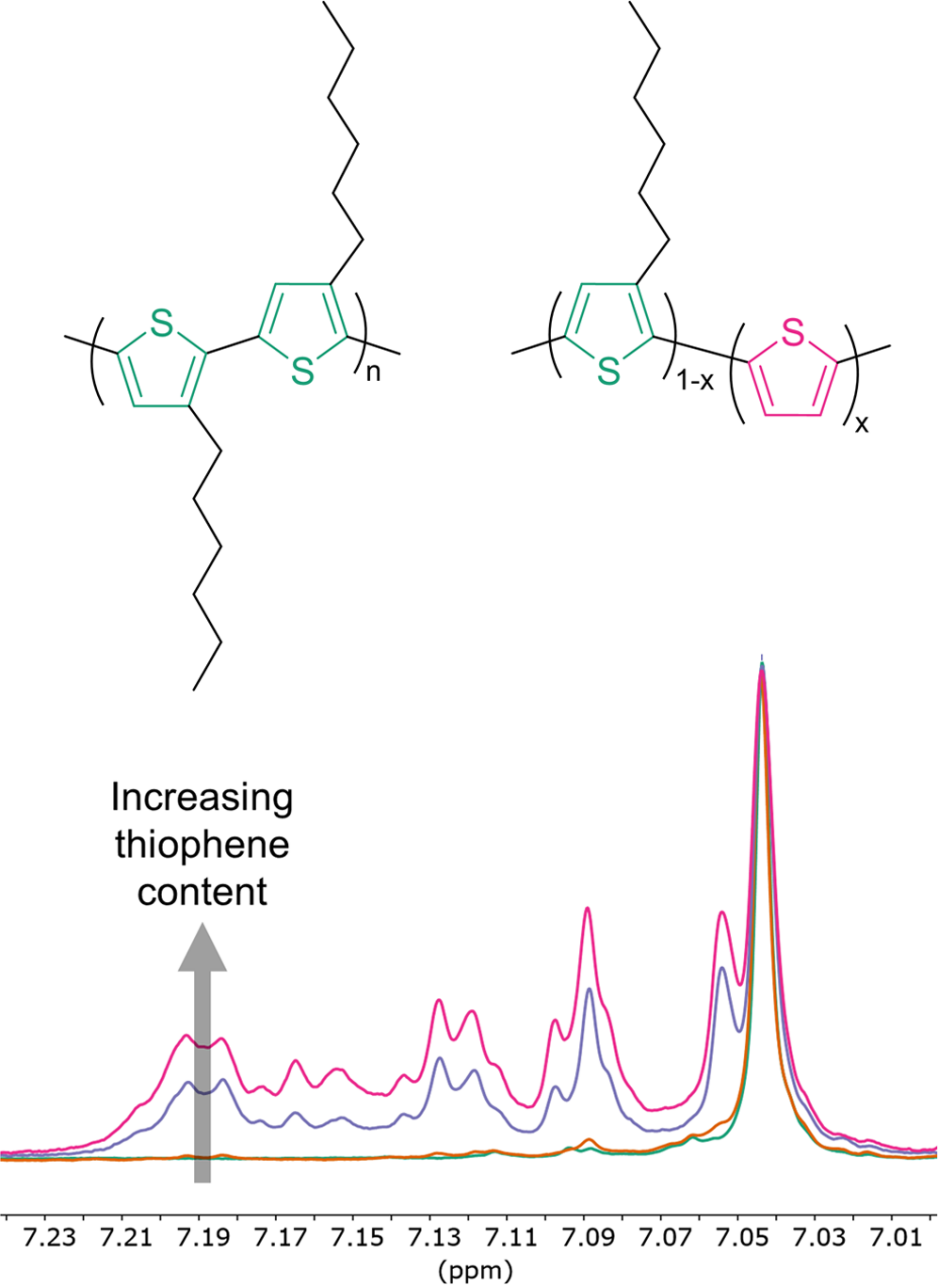
Representation of P3HT (left) and poly(3-hexylthiohene-*ran*-thiophene) (right). Green and pink thiophene units represent monomers with and without side chains, respectively. *x* represents the mol % of unsubstituted thiophene. Superimposed ^1^H NMR spectra of P3HT (green), T_ref_ (yellow), T19 (purple), and T24 (pink) focusing on the aromatic region (bottom).

Herein, we use the benchmark polymer P3HT as a starting point for studying how side chain density can be used as a molecular design parameter for tuning polymer–dopant interactions and the thermoelectric performance of aligned and non-aligned doped polymer thin films. Similar to previous reports, we synthesized three random copolymers comprising the two monomers thiophene and 3-hexylthiophene to afford T*x*, where *x* represents the molar percentage of the unsubstituted thiophene co-monomer in the final polymer (Figure 1).^18^ In other words, T24 means that, on average, for 76 thiophenes bearing a hexyl side chain, there are 24 “naked” thiophenes without side chains. The positions of the units with and without side chains are randomly distributed in the polymer chain. The effect of randomly altering the side chain density in polythiophenes is well documented to improve the charge carrier mobility; however, the effect on the thermoelectric properties is not so established.^19−26^ For the non-aligned films, the electrical conductivity after doping with F4TCNQ increased gradually with the decreasing side chain density, affording a sixfold increase for the highest thiophene content (*x* = 24) compared to P3HT. On the other hand, polymer alignment by high-temperature rubbing proved less efficient with decreasing side chain density.^26^ This is evidenced by decreasing dichroic ratios measured on the aligned films using polarized ultraviolet–visible (UV–vis) spectroscopy. As a consequence, electrical conductivity parallel to the direction of alignment was not clearly correlated to the side chain density due to the simultaneous decrease in melting temperature and lower degree of crystallinity with decreasing side chain density of the neutral polymer films.

## Results and Discussion

### Characterization of the Neutral Polymers

We synthesized the copolymer series comprising thiophene and 3-hexylthiophene co-monomers using the Grignard metathesis (GRIM) method as previously reported (see Supporting Information (SI) Section 2 for details).^18,27^ The regioregularity (RR) of the P3HT reference batch synthesized was estimated to be 90% from high-temperature (90 °C) ^1^H nuclear magnetic resonance (NMR) spectroscopy. We added 10, 20, and 30 mol % 2,5-dibromothiophene to afford our random copolymers with decreasing side chain density. From the ^1^H NMR spectra, we estimated only 19 and 24 mol % unsubstituted thiophene content compared to feed ratios of 20 and 30 mol %, respectively, and therefore named these polymers T19 and T24, respectively (Figure 1 and Table 1). For the 10 mol % feed ratio, however, we calculated ≪1 mol % (see the SI for details), and the ^1^H NMR spectrum closely matches that of P3HT. Yet, increasing the intensity of the spectra reveals new peaks at 7.19 ppm, not observed in the spectrum of P3HT. We therefore conclude that there is a very small amount of unsubstituted thiophene monomer in the polymer; however, it is not significant enough to claim a number. The number-average molar masses measured using size-exclusion chromatography (SEC) are comparable across the copolymer series (∼21–26 kg mol^–1^) and show low dispersity (*Đ*) below 1.8 (Table 1). For P3HT, however, the number-average molar mass is higher (40 kg mol^–1^), and, as a note to the reader, the GPC trace of the P3HT batch used in this study exhibits a bimodal peak (Figure S7 and Table S2). For these reasons, we decided to use the batch with low thiophene content as a reference batch to better compare similar molar masses across the polymer series, consequently naming the polymers as P3HT, T_ref_, T19, and T24.

**Table 1:** Comparison of the Input/Output Thiophene Contents, Molar Masses, and Thermal Characteristics of All Polymers

polymer	output [input] thiophene content (mol %)^a^	*M_n_* (kg mol^–1^) [*Đ*]^b^	*T*_m_/*T*_c_ (°C)^c^
P3HT	0	40 [1.3]	226:192
T_ref_	≪1 [10]	23 [1.5]	221:191
T19	19 [20]	26 [1.8]	187:146
T24	24 [30]	21 [1.5]	146:110

a Output ratio was estimated from ^1^H NMR spectra recorded in d-TCE at 90 °C.

b Number-average molar mass (*M_n_*) was measured by size-exclusion chromatography against polystyrene standards in chlorobenzene at 80 °C. *Đ* = *M*_W_/*M_n_*.

c Obtained by differential scanning calorimetry from the second heating and first cooling cycles recorded under nitrogen at 10 °C min^–1^.

All polymers showed good thermal stability with thermal degradation occurring above 400 °C, determined using thermogravimetric analysis (Figure S8). At lower temperatures, P3HT, T_ref_, T19, and T24 showed endo- and exothermic events ascribed to the melting (*T*_m_) and crystallization (*T*_c_) points, respectively, observed from differential scanning calorimetry (DSC) measurements (Table 1 and Figure S8).^28^ P3HT and T_ref_ exhibited a similar *T*_c_ at around 190 °C, and decreasing the side chain density led to a decrease in *T*_c_.^29^ An exothermic event was also noted just below 100 °C for T_ref_ and T19, not exhibited by P3HT. The same peak might also appear in T24; however, it was not clearly defined due to overlap with the *T*_c_ peak. The thermal results showed that decreasing the side chain density leads to more non-crystalline films due to the decrease in intensity and temperature of the *T*_m_/*T*_c_ peaks.

Solution UV–vis absorbance spectroscopy of P3HT and T_ref_ in chlorobenzene revealed an S_0_ to S_1_ transition at very similar wavelengths around 455 nm, whereas T19 and T24 exhibited a red shift of ∼12 nm (Figure S9). Temperature-dependent UV–vis spectroscopy of the same solutions showed no change in the absorption maxima or shape of the bands when increasing the temperature from 20 to 100 °C, suggesting no aggregation effects. We therefore attribute the red shift observed for T19 and T24 to increased backbone planarity with higher thiophene content, in agreement with previous density functional theory work on related systems.^18,19,30^

**Figure 2:**
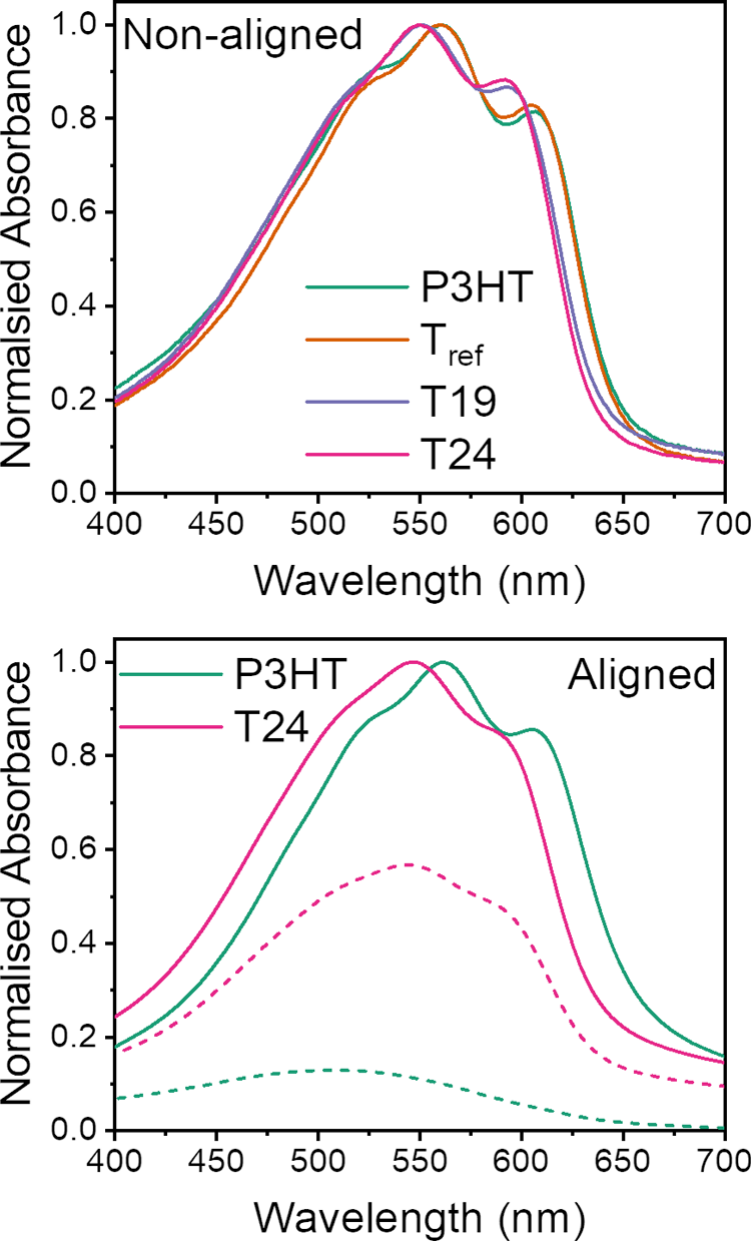
(Top) UV–vis absorbance spectra of P3HT, T_ref_, T19, and T24 films spin-cast from 10 mg mL^–1^*o*-dichlorobenzene (ODCB) solutions at 2000 rpm for 90 s. The spectra were normalized to the lambda max. (Bottom) Polarized UV–vis spectra of aligned P3HT and T24 films in the parallel (solid line) and perpendicular (dashed line) directions to the rubbing direction. P3HT and T24 films were rubbed at 220 and 80 °C, respectively. The transitions in the parallel and perpendicular direction to the rubbing direction were normalized to the lambda max of the transition in the parallel direction.

The energetics and microstructural changes in the solid state were investigated using UV–vis absorbance spectroscopy on thin films spin-cast from ODCB solutions. The spectra of P3HT and T_ref_ revealed one absorbance band arising from the S_0_ to S_1_ transition, exhibiting three shoulders at 606, 561, and 528 nm, ascribed to the 0–0, 0–1, and 0–2 vibronic transitions, respectively (Figure 2 and Table 2).^31,32^ The decreased side chain density in T19 and T24 resulted in an ∼10 nm blue shift of the thin-film UV–vis absorption maxima, coinciding with a more intense 0–0 vibronic peak. The more intense 0–0 transition indicated an increased planarity of the polymer backbone and intrachain coupling, agreeing with the solution UV–vis spectra. We tentatively ascribed the unexpected blue shift to a modified dielectric environment when fewer side chains are present. High-temperature rubbing was used to further assess the effect of decreasing side chain density. Polymer films bar-coated onto sodium polystyrenesulfonate (NaPSS)-coated glass slides from 10 mg mL^–1^ ODCB solutions were mechanically rubbed using a microfibre cloth attached to a rotating cylinder while heating the substrate on a hot plate. Using polarized UV–vis spectroscopy, the degree of alignment characterized by the dichroic ratio (DR) was estimated from the absorbance at 610 nm parallel and perpendicular to the rubbing directions. It has been extensively discussed in the literature that high-temperature rubbing aligns the P3HT crystallites parallel to the rubbing direction, increasing the vibrationally structured absorption band associated with ordered polymer chains.^17,33−35^ On the other hand, the featureless, blue-shifted optical transition in the perpendicular direction to the rubbing direction arises from the absorption of non-aligned disordered polymer chains. We found that decreasing the side chain density lowered the in-plane alignment achieved by high-temperature rubbing, clearly seen when comparing the maximum dichroic ratio at 610 nm from P3HT to T24 (Figure 2, Table 2, and Figure S11). Also, the similarity of the shape in the absorption peaks between the two directions showed that there are also ordered chains in the direction perpendicular to the rubbing direction. In addition, the temperature range, over which optimal alignment occurs, decreased substantially with a lower side chain density. While T_ref_ behaved essentially as pure regioregular P3HT (as expected from the low unsubstituted thiophene content confirmed experimentally, Table 1) and could be rubbed at high temperatures (180 °C), T24 displayed a very different thermomechanical behavior. According to the polarized UV–vis spectra, the highest alignment of T24 occurred around 80 °C with a modest dichroic ratio of 3 compared to a dichroic ratio of 16 for P3HT rubbed at 220 °C. Alignment continuously decreased for T24 films rubbed at higher temperatures, and the films fully delaminated from the substrate at temperatures above 150 °C.

**Table 2:** Optical and Energetic Properties of the Neutral Polymers

polymer	λ_max_ (nm)		c*E*_g_^opt^ (eV)	dDR [rubbing temperature] (°C)]	e*E*_onset_^(ox)^ vs Fc/Fc^+^ (V)	fΦ_wf_ (eV)
	solution^a^	thin film^b^				
P3HT	455	561	1.91	16 [220]	0.05	4.65
T_ref_	456	560	1.91	10 [180]	0.06	4.67
T19	469	550	1.94	6 [150]	0.03	4.71
T24	471	549	1.95	3 [80]	0.03	4.70

a Measured by UV–vis absorbance spectroscopy from 0.01 mg mL^–1^ polymer solutions in chlorobenzene set at 30 °C using a Peltier cooler.

b Measured by UV–vis absorbance spectroscopy from thin films spun from 10 mg mL^–1^ ODCB solutions at 80 °C at 2000 rpm for 90 s onto glass slides.

c Estimated from the optical onset of absorbance. Energy (eV) = 1240 (eV nm)/λ (nm).

d Dichroic ratio was calculated using DR = *A*_∥_/*A*_⊥_.

e Measured by cyclic voltammetry from the onset of oxidation of polymer thin films drop-cast onto a glassy carbon electrode from 1 mg mL^–1^ chloroform solutions in acetonitrile with 0.1 M tetrabutylammonium hexafluorophosphate as the supporting electrolyte. A platinum wire, carbon electrode, and Ag/Ag^+^ electrode were used as the counter, working, and reference electrodes, respectively. Fc/Fc^+^ = Ag/Ag^+^ – 0.115 V.

f Work function measured using PESA on two polymer thin films doctor-bladed onto ITO-coated glass from 10 mg mL^–1^ solutions in chlorobenzene.

**Figure 3:**
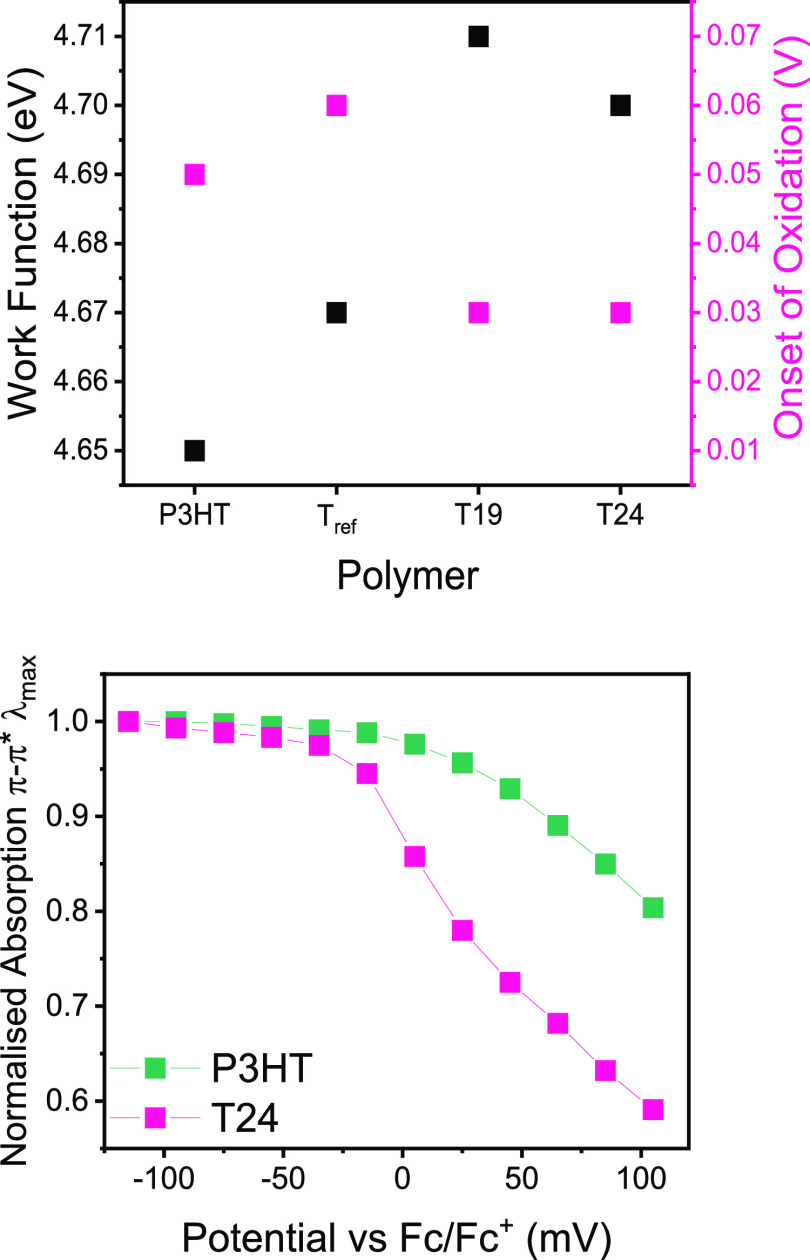
(Top) Plot showing the correlations between work function and the onset of electrochemical oxidation across the polymer series. The work function was obtained via the average of two PESA measurements on 10 mg mL^–1^ ODCB solutions doctor-bladed onto ITO substrates, and the onset of oxidation was taken from the onset of the minor oxidation peak from the CV measurements at a scan rate of 50 mV s^–1^. (Bottom) Plot of the decrease in absorption of the π–π* transition of P3HT and T24 thin films spin-cast from 10 mg mL^–1^ ODCB solutions onto ITO-coated glass with increasing electrochemical potential. Each spectrum was taken 1 min after the applied potential. The trend was normalized to the λ_max_ of the first spectrum taken in the undoped state.

We studied the solid-state electrochemical properties across the polymer series using cyclic voltammetry (CV) and spectroelectrochemistry on non-aligned films (Figures 3, S12, and S14). P3HT and T_ref_ thin films drop-cast onto the carbon working electrode exhibited major electrochemical oxidation waves with peak currents at 0.5 and 0.4 V vs Fc/Fc^+^, respectively, along with minor oxidation waves with onsets at 0.05 V for both films when measuring at a scan rate of 50 mV s^–1^.^11^ Cyclic voltammograms of T19 and T24 revealed both these oxidative waves shifting to lower potentials, thereby indicating slightly smaller ionization potentials by ∼30 meV. We found this unexpected as removal of the side chains would increase the ionization potential due to the loss from the inductive effect, increasing the backbone electron density. We also measured the work function of thin films using photoelectron spectroscopy in air (PESA) and found a ∼40 mV increase in energy upon incorporating >19 mol % thiophene content (Figure 3). Although the variations were small and possibly within the uncertainty of the measurements, the discrepancy between the CV and PESA techniques could arise due to measuring ion insertion into the film vs photoelectron emission, respectively, where the former is largely dependent on morphology.^36^ We also observed a decrease in ionization potential between P3HT and T24 thin films spin-coated onto indium tin oxide (ITO)-coated glass using spectroelectrochemistry (Figure 3). A steady decrease in absorbance of the λ_max_ for the neutral polymer vs potential was seen for P3HT thin films, leading to an onset at +20 mV vs Fc/Fc^+^, whereas a sharp decrease was observed for T24, resulting in an onset at −22 mV vs Fc/Fc^+^. The sharper decrease of the neutral band with increasing potential observed for the novel polymer is of interest for electrochemical devices relying on the modulation of properties upon voltage application, such as electrochromic devices and organic electrochemical transistors (OECTs).

To assess the effect of side chain density on the ordered structures within the solid state, we carried out diffraction on the aligned and non-aligned films. Grazing incidence X-ray diffraction (GIXRD) carried out on non-aligned drop-cast films of P3HT and T_ref_ in the out-of-plane direction showed similar diffraction patterns with the peak at 2θ = 5.5° characteristic of the (*h*00) lamellar stacking crystallites in edge-on orientation (Figure S19).^37^ The stacking distance *d*_100_ was calculated to be 16.3 Å for P3HT and T_ref_, indicating a similar crystal structure. On the other hand, diffraction patterns of T19 and T24 films showed much lower intensity of the (*h*00) peak, resulting from lower structural order. Much smaller *d*_100_ values of 15.2 and 14.6 Å were also observed for T19 and T24 films, respectively. The appearance of a small peak at 2θ = 24.0° for T24 films, with an associated *d*-spacing of 3.7 Å that we assigned to (020) π–π stacking, also suggests a mix of edge-on and face-on orientations of the crystallites.

In rub-oriented films, decreasing the side chain density along the backbone again resulted in a lattice contraction in the (*h*00) direction, observed via electron diffraction, albeit smaller than that observed for the non-aligned films. Most importantly, rubbed T24 thin films displayed no evidence of (*h*02) reflections from the electron diffraction patterns, further confirming a lack of long-range structural order in the stacking of T24 chains. In fact, the overall structure in aligned T24 films is alike the smectic-like phase observed for regioregular P3HT when rubbed at a rubbing temperature of 100 °C.^33,38^

We also observed a reduction in average surface roughness with decreasing side chain density of non-aligned films via atomic force microscopy (AFM) (Figure S20). P3HT and T_ref_ exhibited rough surfaces with average roughness values of 2.9 and 3.5 nm, respectively, whereas T19 and T24 had smoother surface morphologies with average roughness values of 1.8 and 0.8 nm, respectively.

**Figure 4:**
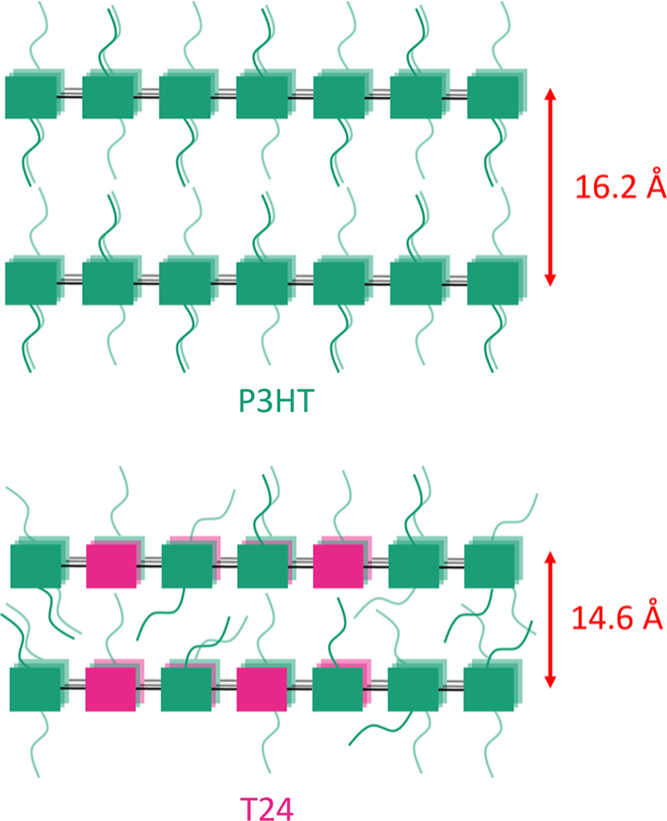
Schematic suggesting how the side chains fill empty space between the polymer backbones for T24 compared to P3HT, interpreted from the diffraction data of the non-aligned films. Green and pink squares represent thiophene monomers with and without a hexyl side chain, respectively.

To summarize, decreasing the side chain density induces a lower degree of long-range order for both aligned and non-aligned films along with a contraction in the (*h*00) lattice parameter. Based on this evidence, we hence speculate that systematic removal of the side chains leads to the vacancies being filled by side chains on neighboring monomers on the same polymer chain, therefore reducing the spacing between the backbones (Figure 4).

To investigate the effects of side chain vacancies on charge transport properties, we fabricated bottom-gate, top-contact organic field-effect transitions (OFETs) with non-aligned undoped polymer channels (refer to the SI for details). All of the polymers across the series showed p-type charge transport as expected. P3HT exhibited a saturation hole mobility of 1.1 × 10^–4^ cm^2^ V^–1^ s^–1^, and the random copolymers showed slightly higher values between 2 and 4 × 10^–4^ cm^2^ V^–1^ s^–1^, tentatively ascribed to increased polymer chain connectivity through localized aggregates, as discussed by Son et al. (Figure S26).^18^ However, the charge carrier mobilities reported here did not reflect the large improvement observed in the previous literature upon removing ∼30% alkyl side chains in polythiophenes, and we believed that the observed differences in our study are not significant.

### Thermoelectric Properties of the Doped Polymers

**Figure 5:**
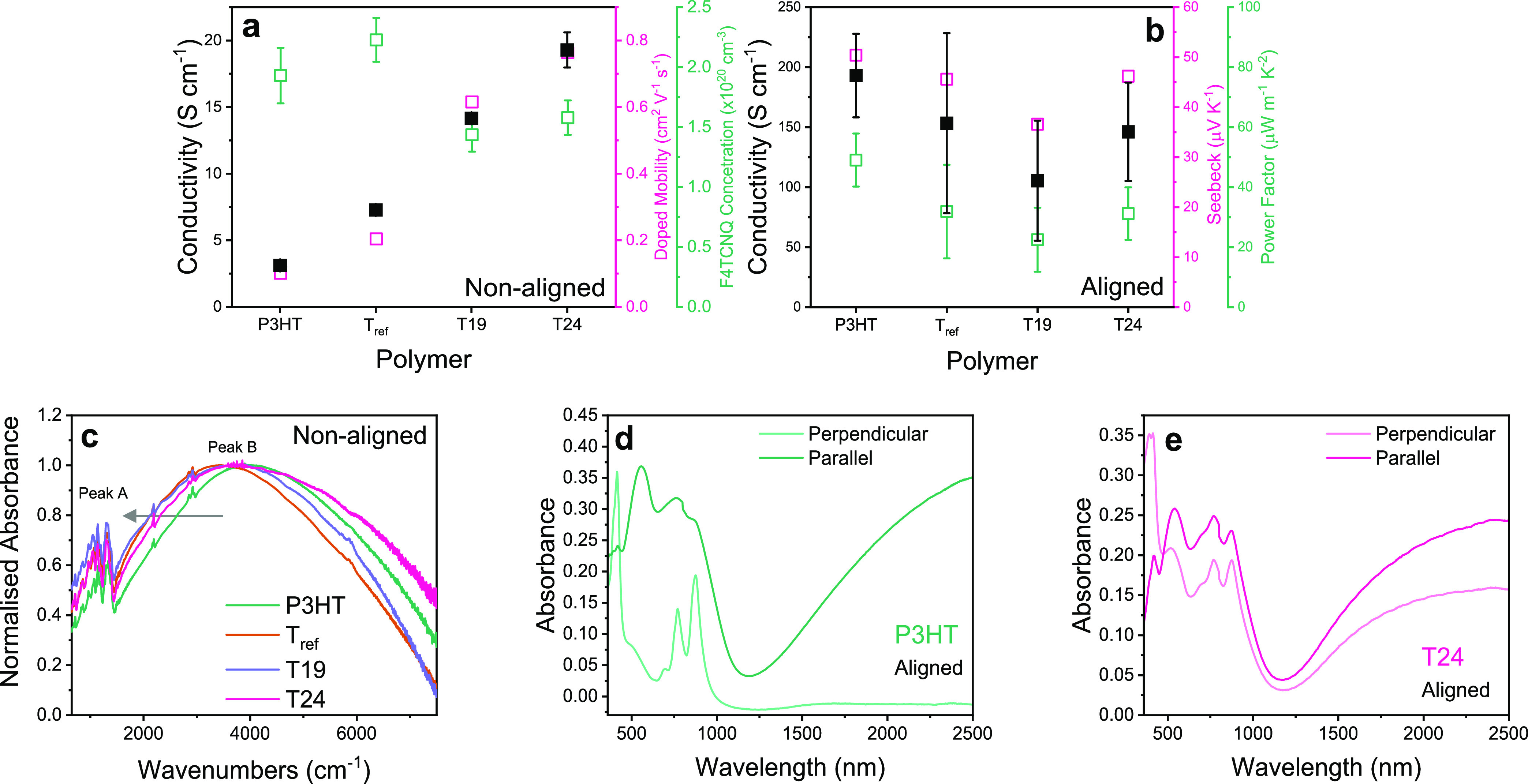
(a) Plot showing the correlation between the measured electrical conductivity (black filled data points), estimated F4TCNQ radical anion concentration (pink filled), and estimated doped mobility (green open) for non-aligned P3HT and copolymer thin films doped with F4TCNQ using HighSqP doping levels. The error bars arose from one standard deviation over three thickness measurements on one film and were propagated through the van der Pauw equation. (b) Plot showing the correlation between the electrical conductivity (black filled), Seebeck coefficient (pink open), and power factor (green open) in the parallel direction to the rubbing direction of aligned films doped with F4TCNQ at 1 mg mL^–1^. The error bars in electrical conductivity arose from one standard deviation over two measurements of two devices. The error bar in the power factor was propagated through PF = σ*S*^2^, where σ and *S* are the electrical conductivity and Seebeck coefficient, respectively. See Supporting Information Section 14 for thickness calculations. (c) FTIR spectra of doped P3HT and copolymer non-aligned films. The arrow indicates to the reader the shift in the peak at ∼4000 cm^–1^. (d, e) UV–vis–NIR spectra of P3HT (d) and T24 (e) aligned films doped with F4TCNQ using ICD of up to 1 mg mL^–1^ measured in the directions perpendicular and parallel to the rubbing direction, respectively.

To explore the electrical properties of the doped polymer series, we doped non-aligned and aligned thin films with the widely studied oxidant F4TCNQ.^39^ We doped the non-aligned films by the sequential processing (SqP) technique using a combination of good and poor solvents, which led to high and low doping levels, which we labeled as HighSqP and LowSqP, respectively (refer to the SI for details).^35,40−42^ P3HT films doped with F4TCNQ under HighSqP and LowSqP conditions exhibited the lowest electrical conductivities in the series with values of around 3.1 and 0.3 S cm^–1^, respectively. With a decreasing side chain density, we found that the conductivity increased gradually to a maximum value of 19.3 S cm^–1^, observed for HighSqP-doped T24 films (Figure 5a). Interestingly, T_ref_ films doped under both conditions showed more than double the conductivity of P3HT, although T_ref_ displayed nearly identical structural order in the neutral films. To ensure the validity of our claims, we also measured the conductivity of a P3HT batch with higher molar mass and regioregularity doped with F4TCNQ under the same conditions (Table S7). We also found that T19 and T24 exhibited higher electrical conductivity under the same doping conditions compared to this better-performing batch of P3HT.

Turning our attention to the aligned films, we measured the conductivity parallel and perpendicular to the rubbing direction upon doping with 0.1–1 mg mL^–1^ F4TCNQ solutions in acetonitrile using the incremental concentration doping method (ICD) (Figure 5b).^35^ In agreement with previous literature on highly orientated polymer films, the measured electrical conductivity of P3HT and the copolymers films was much higher (∼by 1 order of magnitude) in the parallel direction to rubbing compared to that in the perpendicular direction (Figure S30). Alignment of the crystallites parallel to the rubbing direction improves the charge carrier mobility in that direction. As a note here to the reader, we did not use the same doping conditions as above, as we were exploring the effects of high-temperature rubbing not maximizing performance. At the highest F4TCNQ concentration (1 mg mL^–1^) in the parallel direction, P3HT exhibited the highest conductivity of 193 S cm^–1^, not T24, contrary to the trend observed with the non-aligned films. The conductivity variation of the doped films as a function of side chain percentage reflects mainly the limited thermomechanical properties of the polythiophene with a low side chain density. We subsequently determined the Seebeck coefficients of the aligned doped films, allowing us to estimate the power factor and hence evaluate the potential of the synthetic strategy proposed here for the design of semiconducting polymers for thermoelectric applications. At 1 mg mL^–1^ dopant concentration, the Seebeck coefficients in the parallel direction follow the same trend as the electrical conductivity, leading therefore to the power factor in the parallel direction also following the same trend (Figure 5b). Regardless of the structure of the polymer and the trend observed in non-aligned films, the conductivity and Seebeck coefficients parallel to the rubbing direction of the aligned films appeared to loosely follow the degree of alignment (quantified by the dichroic ratio, Table 2). We further explored the large improvement in conductivity observed in the non-aligned films and where the discrepancy between the aligned and non-aligned films arises from, as discussed below.

### Characterization of the Doped Films

UV–vis absorbance spectroscopy carried out on the doped non-aligned films revealed partial bleaching of the neutral band assigned to the polymer oxidation and four new peaks at 420, 697, 770, and 855 nm that are characteristic of the F4TCNQ radical anion, confirming that integer charge transfer (ICT) has occurred (Figures S27 and S33–S36).^43,44^ We fitted the absorbance spectra with Gaussians to estimate the F4TCNQ radical anion concentration and found that the density of ionized F4TCNQ is very similar for both HighSqP (∼1.8 × 10^20^ ± 20% cm^–3^) and LowSqP (∼1.0 × 10^20^ ± 12% cm^–3^) regardless of the thiophene content (Figure 4 and Table S8). This suggests that the significant improvement in conductivity of the non-aligned films did not arise from a larger charge carrier concentration in the doped films but rather from an increase in the charge carrier mobility. Under the strong assumption that each F4TCNQ radical anion induces a mobile carrier, we estimated the charge carrier mobility within the doped films by dividing the measured conductivity by the calculated F4TCNQ radical anion density (σ = μ·*n*). We found that under HighSqP conditions, the estimated doped mobility increased significantly with decreasing side chain density. The maximum carrier mobility is hence found for doped T24 non-aligned films to be four times larger than that of doped P3HT films (Figure 5a).

To confirm the improvement of carrier mobility with decreasing side chain density, we probed the changes in the P1 polaron peak across the polymer series using Fourier transform infrared (FTIR) spectroscopy on doped non-aligned films (Figure 5c). From the normalized spectra, a red shift of the large band at ∼4000 cm^–1^ (peak B) coinciding with the increased intensity of the peak hidden by the so-called IR-active vibrations (IRAVs) observed below 1500 cm^–1^ (peak A) indicated stronger inter- and intrachain coupling in ordered polymers.^45,46^ Under HighSqP doping conditions, peak B red-shifted further and peak A increased in intensity for doped T_ref_, T19, and T24 films compared to doped P3HT films, pointing toward an even higher degree of polaron delocalization. We note here that the broadening of peak B suggests a larger distribution of localized and delocalized polarons compared to P3HT. These measurements suggest more uniform doping of both ordered and disordered regions in T19 and T24, rationalizing the increased conductivity and mobility in these samples.^47^

To investigate the origin of the improved conductivity in the non-aligned films with decreasing side chain density, we measured the out-of-plane X-ray diffraction patterns of the drop-cast non-aligned films doped by immersion in 2 mg mL^–1^ F4TCNQ solutions in acetonitrile overnight. The *d*_100_ lamellar distance for P3HT and T_ref_ films increased by ∼2.5 Å from 16.3 Å in the neutral state to 18.8–18.9 Å in the doped state due to dopant insertion between the polymer backbones in the side chain region (Table S5).^48^ T19 and T24 films also exhibited a lamellar expansion of ∼2.7 Å upon dopant insertion and oxidation of the polymer backbone but retained a smaller *d*_100_ compared to P3HT and T_ref_ (17.5–17.7 Å). Due to instrument capabilities, we only measured the out-of-plane direction; however, we expected a parallel contraction in the π-stacking direction, as previously reported.^27,37^ Concurrent with a smaller *d*_100_, doped T19 and T24 films exhibited larger lamellar coherence lengths (∼133 Å) compared to doped P3HT and T_ref_ films (∼109 Å), demonstrating that doping the non-aligned films with lower side chain density induced higher long-range ordering. Comparing the X-ray diffraction patterns of the neutral and doped non-aligned films, the results suggest that lowering the side chain density reduces the order in the neutral state compared to P3HT; however, upon doping, there are more ordered domains. This hypothesis agrees with the improved measured electrical conductivity and higher calculated charge carrier mobility of the doped films when decreasing the side chain density.

Turning our attention to the doped aligned films, we used polarized UV–vis–NIR absorbance spectroscopy in the directions parallel and perpendicular to the rubbing direction. Aligned P3HT thin films displayed the F4TCNQ radical anion peaks in the perpendicular direction and the neutral and polaronic transitions in the parallel direction to the rubbing direction (Figure 5d). These findings agree with previous literature, indicating that the F4TCNQ radical anions sit perpendicular to the oriented polymer backbone and that the films retain their anisotropy despite dopant insertion.^35^ Doped aligned T_ref_ films showed almost identical spectra to P3HT, suggesting a similarly high degree of alignment, again corroborating the strong similarity of P3HT and T_ref_ (Figure S34). However, all of the optical transitions from both the polymer and the dopant are clearly observed in both the parallel and perpendicular directions for aligned and doped T19 and T24 films (Figures 5e and S35). The lack of anisotropy indicates a low degree of alignment of polymer backbones in the doped state. This likely arises from the relatively low dichroic ratio of undoped films as discussed above.

**Figure 6:**
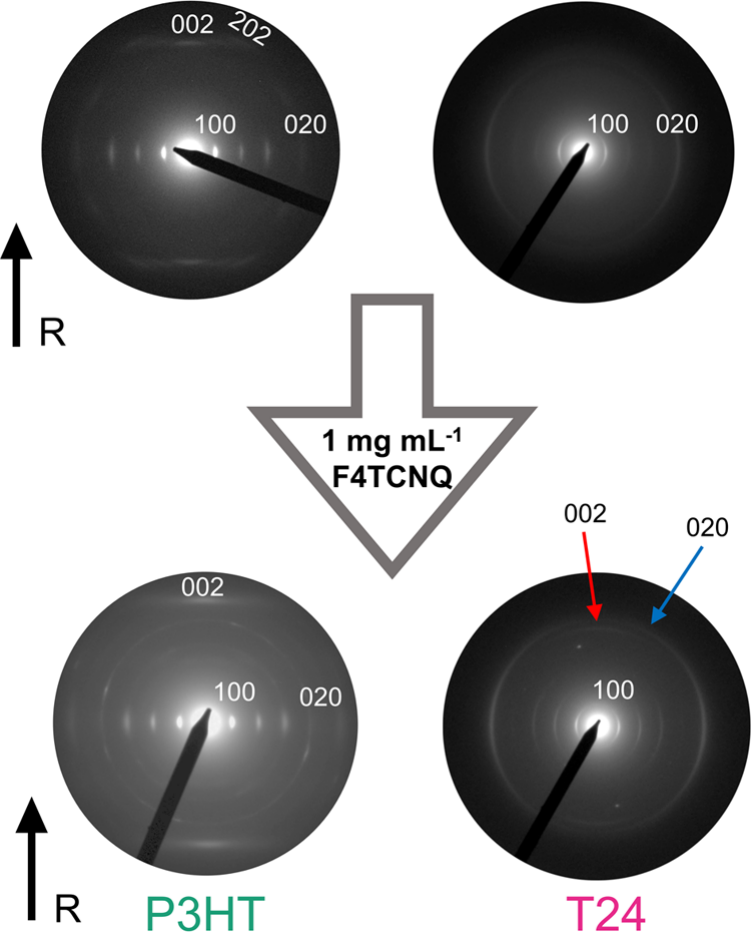
Electron diffraction patterns of aligned P3HT and T24 films before and after doping with F4TCNQ at a concentration of 1 mg mL^–1^. The ‘R’ arrows represent the rubbing direction. The red and blue arrows direct the reader to the (002) and (020) reflections after doping to 1 mg mL^–1^ for aligned T24 films.

Electron diffraction using transmission electron microscopy (TEM) was used to probe the evolution of the microstructure as a function of increasing the doping concentration on doped aligned films (Figures 6 and S37). The impact of doping on the structure of T_ref_ films is almost identical to that observed for P3HT, further confirming the similarity between the two, where doping preserves the in-plane (edge-on) orientation of polymer chains. Electron diffraction showed the crystal lattice expanding in the side chain (lamellar) direction with an increase of *d*_100_ from 16.2 to 18.2 Å for aligned P3HT and T_ref_ films doped with 1 mg mL^–1^ F4TCNQ solutions, whereas the π-stacking periodicity (*d*_020_) reduces from 3.7 to 3.6 Å. As observed previously for aligned regioregular P3HT films, doping results in a notable change of intensity of the (*h*0l) lamellar stacking reflections (*l* = 0, 1, 2). The expansion of the (100) reflection and contraction of the (020) reflection also support the results from the GIXRD measures of the non-aligned films, suggesting similar crystal structures between aligned and non-aligned films upon doping. The (002) end-to-end translational reflection becomes predominant with a total loss of intensity for (102) and (202) reflections, and as demonstrated in our previous work on F4TCNQ doping of P3HT, this change in the electron diffraction pattern is a fingerprint of dopant intercalation into the crystal lattice of P3HT.^49^

For the two other polymers, T19 and T24, doping with 1 mg mL^–1^ F4TCNQ solutions induced similar lattice modifications in the sense that the unit cell expanded in the side chain direction and contracted in the π-stacking direction. Compared to P3HT and T_ref_, however, the lattice parameter reached saturation at a concentration of 0.1 mg mL^–1^ F4TCNQ solutions for aligned T19 and T24 films. Markedly, structural reorganization in the aligned mesophase of T24 upon doping was observed more strongly than that of P3HT using electron diffraction, where the (002) reflection became visible after doping (Figure 6, red arrow). However, the appearance of this reflection for doped T24 films signifies a reorganization of polymer chains within the π-stacks, such that the successive thiophene backbones shift in the chain axis direction to create some cavities to host the dopant molecules in the crystal lattice of the polymer. The same mechanism was observed for all polymers, regardless of the amount of unsubstituted thiophene in the backbone. However, it is possible that with an increasing percentage of unsubstituted thiophene, the cost of reorganization of the backbones within π-stacks was reduced, and therefore, the effect is observed more strongly with decreasing side chain density.

## Conclusions

Using the benchmark semiconducting polymer P3HT as our model system, we systematically studied how the side chain density influences the optical and electronic properties of non-aligned and aligned films in view of thermoelectric applications. A simple synthetic protocol provides low-cost access to thiophene and 3-hexylthiophene random copolymers with varying contents of unsubstituted thiophenes (and thus side chain vacancies) by controlling the monomer feed ratios. Despite a 10 mol % feed ratio, polymer T_ref_ actually showed <1 mol % side chain vacancies, making it structurally very similar to P3HT, thus resulting in very similar optical and electronic properties to P3HT. Increasing the unsubstituted thiophene content to 20 and 30% afforded polymers T19 and T24, respectively, with better agreement between feed ratios and observed degrees of side chain vacancies. For the non-aligned polymer films, the electrical conductivity after doping with F4TCNQ increased markedly with decreasing side chain density; a more than sixfold increase from 3.1 to 19.3 S cm^–1^ was observed for T24 compared to P3HT under high-doping conditions. While the F4TCNQ radical anion concentration remained fairly constant across the copolymer series upon doping, a significant increase in the estimated doped charge carrier mobility was observed for the copolymers with lower side chain densities. A recent study by Kim et al. has also observed the same trend upon doping a similar copolymer system with F4TCNQ. Albeit at lower electrical conductivity, they observed an increase in electrical conductivity of thin films with more thiophene co-monomer when doping via blending in solution.^25^

As a simple synthetic tool, we thus find that introducing side chain vacancies does not, perhaps somewhat contrary to simplistic consideration regarding the extra free volume, allow for higher charge carrier concentrations during doping. On the other hand, the introduction of side chain vacancies makes the T19 and T24 copolymers more facile to structural reorganization upon doping. This is evidenced by our electron diffraction studies, where the (002) reflection of T24 becomes visible after doping, and by X-ray diffraction studies, where longer crystallite coherence lengths are measured for the doped T24 films compared to those for doped P3HT films. These findings are corroborated by the higher charge carrier mobilities extracted for the doped films with lower side chain densities, which ultimately lead to the observed trend of increasing electrical conductivity with decreasing side chain density.

However, thermal rubbing proved less efficient with decreasing side chain density, as evident from decreasing dichroic ratios measured on the aligned films using polarized UV–vis absorbance spectroscopy. Therefore, electrical conductivity parallel to the direction of alignment was not clearly correlated to the degree of side chain vacancies due to the simultaneous change in the melting temperature of the polymers. Although decreasing the side chain density allowed for a beneficial reorganization of the non-aligned films upon doping, these attributes prevent a high degree of structural alignment by thermal rubbing. That being said, aligned doped T24 films showed a comparable power factor (31 μW m^–1^ K^–2^) to P3HT (49 μW m^–1^ K^–2^) even with a very low dichroic ratio.

Our results therefore suggest that side chain engineering, and in particular introducing unsubstituted monomer units, can be an effective way to fine-tune the degree of order/disorder of the conjugated polymer to suit the intended application. This simple synthetic method could lead to low-cost materials suited to cheap large-scale device fabrication techniques, i.e., roll-to-roll printing. Although our initial attempts at alignment prior to doping do not lead to enhanced electrical conductivity, we anticipate that further optimization of dichroic ratio with low side chain density could afford further improvements.

## Experimental Section

### Materials

3-Hexylthiophene and dichloro(1,3-bis(diphenylphosphino)propane)nickel (Ni(dppp)Cl_2_) were purchased from Fluorochem. *N*-bromosuccinimide (NBS) and isopropyl magnesium chloride lithium chloride solution (1.3 M in THF) were purchased from Sigma-Aldrich. 2,5-Dibromothiophene was purchased from Tokyo Chemical Industry. 2,3,5,6-Tetrafluoro-7,7,8,8-tetracyanoquinodimethane (F4TCNQ) was purchased from Ossila or Sigma-Aldrich. CDCl_3_ and deuterated tetrachloroethane (d_2_-TCE) were purchased from Cambridge isotopes. Dry tetrahydrofuran (THF) (99.5% over molecular sieves with AgroSeal) and dry dimethylformamide (DMF) (99.5% over molecular sieves with AgroSeal) were purchased from Acros Organics. Ortho-dichlorobenzene (ODCB) was purchased from either Acros Organics or Sigma-Aldrich. Unless stated, all other solvents were HPLC-grade purchased from Honeywell. All chemicals were used as purchased without further purification. Details of the synthesis of P3HT, T_ref_, T19, and T24 and NMR characterization are described in the Supporting Information.

### General Experimental Details

GPC was performed on a Shimadzu Prominence GPC system using chlorobenzene as the mobile phase at a flow rate of 1 mL min^–1^. The molar mass of each polymer was measured against polystyrene standards. Thermogravimetric analysis (TGA) was carried out on a TA Instruments Q500 under N_2_ at 10 °C min^–1^. DSC was performed using a TA Instruments DSC25 under N_2_ at 10 °C min^–1^. Unless mentioned, thin films were fabricated from spin-coated 10 mg mL^–1^ ODCB solutions at 2000 rpm for 90 s and then 8000 rpm for 30 s. UV–vis spectroscopy of the non-aligned and aligned films was performed on a Shimadzu UV3600 UV–vis–NIR spectrometer and a Varian Cary5000 spectrometer. FTIR spectroscopy was performed on a Bruker Vertex 70v FTIR spectrometer under a vacuum using a DLaTGS detector. CV experiments were conducted using a Palm EmStat3 with Ag/Ag^+^, platinum, and glassy carbon as the reference, counter, and working electrodes, respectively. Chloroform solutions (1 mg mL^–1^) of each polymer were drop-cast onto the working electrode. Tetrabutylammonium hexafluorophosphate (0.1 M) in N_2_-degassed acetonitrile was used as the supporting electrolyte. Spectroelectrochemical experiments were performed using the same setup, but thin films of P3HT and T24 were spin-coated onto ITO substrates as the working electrode, and the UV–vis spectra were measured using the Shimadzu UV3600 UV–vis–NIR spectrometer. PESA was performed on polymer thin films, doctor-bladed from 10 mg mL^–1^ ODCB solutions onto ITO-coated glass, and measured using an AC-2 Model from Riken Instruments. GIXRD was carried out on drop-cast films from 20 mg mL^–1^ ODCB polymer solutions onto silicon substrates and measured using a PANalytical X’Pert Pro diffractometer. AFM was carried out on polymer thin films using a Bruker Dimension Icon System. ScanAsyst Air tips were used to image the samples in PeakForce Quantitative Nanomechanical Property Mapping (QNM) mode. TEM ED patterns were obtained using a CM12 Philips microscope equipped with an MVII (Soft Imaging System) camera on polymers films coated with a thin amorphous carbon film and floated onto a TEM copper grid.

### OFET Device Fabrication

The backgate and gate dielectrics were chosen to be highly n-doped silicon and thermally grown SiO_2_ (300 nm), respectively. After cleaning Si/SiO_2_ substrates with oxygen plasma at 300W for 10 min, the substrates were then immersed in 3 wt % PTS/toluene solution for 15 h at 90 °C. The excess PTS on Si/SiO_2_ substrates was cleaned by sonication with toluene, followed by rinsing with toluene, acetone, and isopropanol. The polymer solutions were preheated at 80 °C for 1–2 h before film deposition. The polymers (10 mg mL^–1^) in ODCB were spin-coated on PTS-functionalized Si wafers. The Cr/Au electrodes (5/250 nm) were thermally evaporated under high vacuum (10^–6^ mbar) as the source and drain electrodes (*W*/*L* = 1000:20). The prepared bottom gate top contact OFETs were placed in a nitrogen glovebox prior to testing.

### Polymer Thin Film Alignment

The aligned polymer films were prepared by doctor-blading a hot solution in an ODCB solution (10 mg mL^–1^) at 160 °C on cleaned glass slides covered with a sacrificial polymer film of water-soluble NaPSS (10 mg mL^–1^ aq). The orientation of the films by high-temperature rubbing followed the protocol described in previous publications.^33,50^ Rubbing was performed by using a homemade setup. It consisted of a rotating cylinder covered with a polyester cloth and a translating hot plate.

### Thermoelectric Characterization Details

The electrical conductivity of the F4TCNQ-doped non-aligned polymer thin films was measured on a Karl Suss probe station under a nitrogen atmosphere using an Agilent 4155B source meter following the standard van der Pauw method. The non-aligned films were doped using sequential doping methods with F4TCNQ as the oxidant, as described in the Supporting Information. The electrical conductivity of the F4TCNQ-doped aligned films was measured using a Keithley 2634B source meter and a Lab Assistant Semiprobe station under a nitrogen atmosphere. Aligned thin films were transferred to substrates with gold contacts with a linear four-point probe geometry. The Seebeck coefficient was measured using a differential temperature method on the same devices by varying the temperature gradient across the substrate and measuring the corresponding thermovoltage.

## Supporting Information Available

Synthetic procedures for the monomers and polymers; nuclear magnetic resonance spectra; gel permeation chromatography data; thermal characterization analysis; thin-film and solution UV–vis–NIR spectroscopy of the aligned and non-aligned films in the doped and neutral states; electrochemical and spectroelectrochemical experiments; work function measurements; roughness measurements; transistor fabrication and analysis; conductivity measurements of the aligned and non-aligned films; fittings of the UV–vis spectra of the doped non-aligned thin films; Fourier transform infrared spectra of the non-aligned doped films; X-ray diffraction analysis of the drop-cast thick films; method for producing highly orientated thin films; data extracted from the electron diffraction patterns; and calibration curves used to estimate the thickness of the aligned thin films (PDF)
