# Supplementary material for: Interplay between Side Chain Density and Polymer Alignment: Two Competing Strategies for Enhancing the Thermoelectric Performance of P3HT Analogues
Source: Chem Mater. 2023 Oct 26;35(21):9029–39. doi: 10.1021/acs.chemmater.3c01680 (PMC10653083; doi:10.1021/acs.chemmater.3c01680)
Supplement: Supplementary file 1 — cm3c01680_si_001.pdf [file cm3c01680_si_001.pdf]

Supporting Information

The Interplay Between Side Chain Density and Polymer Alignment: Two Competing Strategies for Enhancing the Thermoelectric Performance of P3HT Analogues

Peter A. Gilhooly-Finn*[†,‡], Ian E. Jacobs[§], Olivier Bardagot[||], Yasser Zaffar[‡], Antoine Lemaire[#], Shubhradip Guchait[#], Lu Zhang[§], Mark Freeley[‡], William Neal[‡], Fanny Richard[††], Matteo Palma[‡], Natalie Banerji[||], Henning Sirringhaus[§], Martin Brinkmann[#] and Christian B. Nielsen*[‡]

[†]Department of Chemistry, University College London, Gower Street, London, WC1E 6BT, UK.

[‡]Department of Chemistry, Queen Mary University of London, Mile End Road, E1 4NS, London, UK.

[§]Optoelectronics Group, University of Cambridge, Cavendish Laboratory, J J Thomson Avenue, Cambridge, UK.

[||]Department of Chemistry, Biochemistry and Pharmaceutical Sciences, University of Bern, Freiestrasse 3, 3012 Bern, Switzerland.

[#]Charles Sadron Institute (ICS), CNRS Université de Strasbourg, UPR 22, 23 rue du Loess, Strasbourg Cedex 02, 67034, France.

[††]Université de Strasbourg, CNRS, ISIS UMR 7006, Strasbourg 67000, France.

Contents

1. Materials.. 3
2. Synthesis.. 3
3. Nuclear Magnetic Spectroscopy (NMR).. 6
4. Gel Permeation Chromatography (GPC) 10
5. Thermal characteristics 11
6. Thin film fabrication 12
7. UV-Vis spectroscopy 12
8. Electrochemistry and Spectroelectrochemical Experiments........................ 14
9. Work Function Measurements.. 16
10. X-ray diffraction... 18
11. Roughness measurements 20
12. Organic Field Effect Transistor (OFET) Measurements............................... 20
13. Conductivity, UV-Vis Absorbance and FTIR Spectroscopy of Doped Non-Aligned Thin Films……………………………………………………………………………23
14. UV-Vis-NIR Spectroscopy, Electron Diffraction and Thermoelectric Characteristics of Doped Aligned Thin Films.. 26
15. References 32

1. Materials

For synthesis, 3-hexylthiophene and dichloro(1,3-bis(diphenylphosphino)propane)nickel (Ni(dppp)Cl_2) were purchased from Fluorochem. N-bromosuccinimide (NBS) and isopropyl magnesium chloride lithium chloride solution (1.3 M in THF) were purchased from Sigma-Aldrich. 2,5-dibromothiophene was purchased from Tokyo Chemical Industry. 2,3,5,6-Tetrafluoro-7,7,8,8-tetracyanoquinodimethane (F4TCNQ) was purchased from Ossila or Sigma-Aldrich. $CDCl_3$ and deuterated tetrachloroethane (d_2-TCE) was purchased from Cambridge isotopes. Dry tetrahydrofuran (THF) (99.5% over molecular sieves with AgroSeal) and dry dimethylformamide (DMF) (99.5% over molecular sieves with AgroSeal) were purchased from Acros Organics. Ortho-dichlorobenzene (ODCB) was purchased from either Acros Organics or Sigma-Aldrich. Unless stated all other solvents are HPLC grade purchased from Honeywell. All chemicals were used as purchased without further purification.

2. Synthesis

Synthesis of 2,5-dibromo-3-hexylthiophene (**1**)

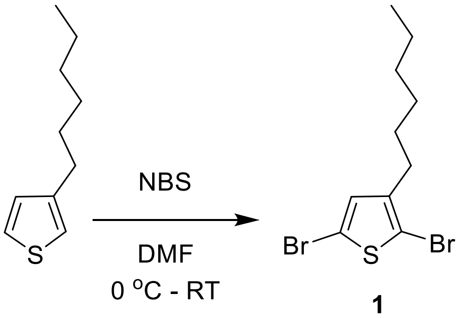

1

NBS (6.98 g, 39.06 mmol, 2.2 eq) was and dissolved in dry DMF (100mL) under a nitrogen atmosphere, covered from light and cooled to 0 °C. To this solution, 3-hexylthiophene (2.99 g, 17.75 mmol) was added dropwise and then stirred overnight, allowing to reach RT (yellow solution). The solution was quenched with H_2O (100 mL) and the product was extracted with ethyl acetate (3 x 80mL). Combined organic layers were washed with H_2O (3 x 100 mL) and brine (100 mL), dried over anhydrous MgSO4. Solvent evaporation and subsequent purification by column chromatography in hexanes (Rf = 0.64) yielded a colourless oil (5.22 g, 16.01 mmol, 90 %) as the product (**1**). 1H NMR (400 MHz, $CDCl_3$) δ 6.78 (s, 1H), 2.50, (t, J = 7.6 Hz, 2H), 1.54 (quin, J = 6.4 Hz, 2H), 1.34 - 1.30 (m, 6H), 0.89 (t, J = 6.6 Hz, 3H). 13C NMR (100 MHz, CDCl3) δ 142, 130, 110, 107, 32, 30, 29, 26, 14, in accordance with literature. [1]

Synthesis of poly(3-hexylthiophene) (90% RR) (**P3HT**)

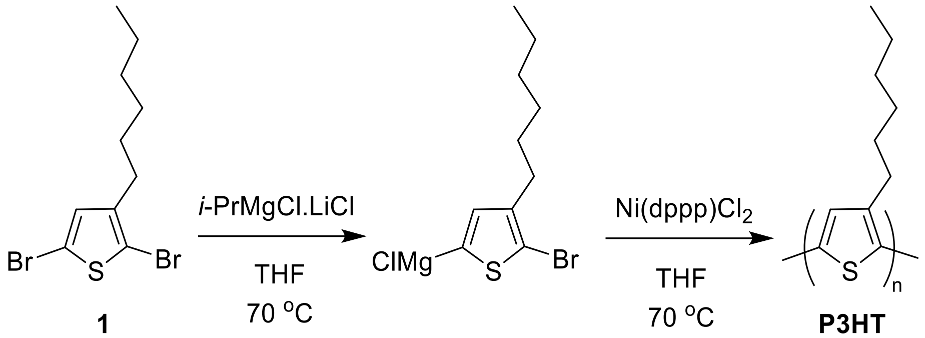

2,5-Dibromo-3-hexyl-thiophene (534 mg, 1.64 mmol) dissolved in dry THF (3.8 mL) in an oven dried microwave vial under nitrogen. It is paramount to ensure all glassware is dry to provide the highest yield. Added isopropyl magnesium chloride lithium chloride complex (1.3 M in THF, 1.2 mL, 0.98 eq) to the solution dropwise and the reaction mixture was stirred for 2 hr at 70 °C (dark yellow solution). This was then added to a suspension of Ni(dppp)Cl_2 (9 mg, 0.02 mmol, 1 mol%) in dry THF (3 mL), in a separate oven dried microwave vial and the polymerisation was stirred at 70 °C overnight. After cooling to room temperature, the reaction was terminated by addition of 1 mL of HCl (10 % (v/v)) followed again by stirring for 20 minutes. The mixture was added to cold well stirred methanol. Obtained precipitates were Soxhlet extracted in methanol, acetone, hexane, and chloroform. The chloroform fraction was concentrated, reprecipitated in methanol and dried under vacuum at 40 °C to give the polymer (181 mg, 68 %) as a brown solid. ^{1}H NMR (400 MHz, $CDCl_3$) δ 6.98 (s, 1H), 2.80 (t, *J* = 7.8 Hz, 2H), 1.71 (quin, *J* = 8.2 2H), 1.45 - 1.33 (m, 6H), 0.91 (t, *J* = 6.9 Hz, 3H). GPC ($CHCl_3$): Mn = 39.7 kg mol^{-1}, Mw = 41.2 kg mol^{-1}, PDI = 1.3.

Synthesis of poly(3-hexylthiophene-*ran*-thiophene) (T_{ref}, T19 and T24)

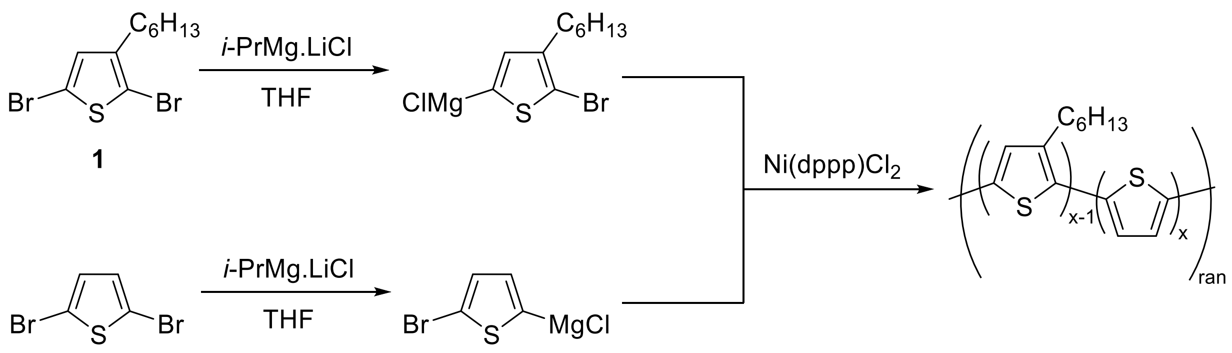

T_{ref} (x << 0.01)

To two separate, oven dried microwave vials, 2,5-dibromothiophene (67.4 mg, 0.28 mmol, 0.1 eq) and 2,5-dibromo-3-hexylthiophene (**1**) (811.8 mg, 2.50 mmol 0.9 eq) were added under nitrogen respectively and each dissolved in dry THF (5 mL). To the 2,5-dibromothiophene solution, isopropyl magnesium chloride lithium chloride (1.3 M in THF, 0.20 mL, 0.27 mmol, 0.95 eq) was added. To the 2,5-dibromo-3-hexylthiophene solution, isopropyl magnesium chloride lithium chloride (1.3 M in THF, 1.82 mL, 2.37 mmol, 0.95 eq) was added and both solutions were heated to 70 ℃ for two hours, with constant stirring. After heating for two hours, the monomer solutions were cooled to 0 ℃ in an ice bath and transferred to the same flask under nitrogen. The polymerisation was initiated through the addition of degassed Ni(dppp)Cl_2 (8.8 mg, 16.24 μmol, 0.6 mol%) dispersed in dry THF (3 mL) to the reaction vessel and heated to 70 ℃ overnight with constant stirring. After cooling to room temperature, the reaction was quenched with 2 mL of HCl (10 % (v/v)) and precipitated into cold methanol. After filtering the solution into a thimble, Soxhlet extraction was carried out using methanol, acetone, hexane, and chloroform. The chloroform fraction was collected, concentrated under reduced pressure, and re-precipitated in cold methanol. The precipitated polymer was vacuum filtered and dried under vacuum at 40 °C giving the polymer (T_{ref}) (259.3 mg, 60 %) as dark purple solid. ^{1}H NMR (400 MHz, d_2-TCE, 363 K) δ 7.04 (s, 1H), 3.00 – 2.50 (m, 2H), 1.78 (quin, J = 7.7 Hz, 2H), 1.52 – 1.41 (m, 6H), 0.98 (t, J = 6.9 Hz, 3H). GCP (CB): Mn = 23.2 kg mol^{-1}, Mw = 34.5 kg mol^{-1}, PDI = 1.5.

T19 (x = 0.19)

Following the polymerisation procedure above with 2,5-dibromothiophene (500.4 mg, 2.07 mmol, 0.2 eq) and 2,5-dibromo-3-hexylthiophene (**1**) (2.70 g, 8.27 mmol, 0.8 eq) with isopropyl magnesium chloride lithium chloride complex (1.3M in THF) added to each (1.5 mL, 1.96 mmol, 0.95 eq and 6.0 mL, 7.85 mmol, 0.95 eq respectively). Added Ni(dppp)Cl_2 (33.9 mg, 62.54 μmol, 1 mol%) to combined solutions to afford product (T19) (1.0 g, 65 %) as a dark purple solid. ^{1}H NMR (400 MHz, d_2-TCE, 363 K) δ 7.19 – 7.04 (m, 1H), 3.00 – 2.50 (m, 2H), 1.78 (quin, J = 6.6 Hz, 2H), 1.52 – 1.41 (m, 6H), 0.98 (t, J = 6.9 Hz, 3H). GPC (CB): Mn = 26.1 kg mol^{-1}, Mw = 46.8 kg mol^{-1}, PDI = 1.8.

T24 (x = 0.24)

Following the polymerisation procedure above with 2,5-dibromothiophene (317.5 mg, 1.31 mmol, 0.3 eq) and 2,5-dibromo-3-hexylthiophene (**1**) (1.0 g, 3.07 mmol, 0.7 eq) with isopropyl magnesium chloride lithium chloride complex (1.3M in THF) added to each (1.0 mL, 1.25 mmol, 0.95 eq and 2.2 mL, 2.92 mmol, 0.95 eq respectively). Added Ni(dppp)Cl_2 (25.0 mg,

46.12 μmol, 1 mol%) to combined solutions to afford product (T24) (399.0 mg, 65 %) as a dark purple solid. ^{1}H NMR (400 MHz, d_2-TCE, 363 K) δ 7.04 (s, 1H), 3.00 – 2.50 (m, 2H), 1.78 (quin, J = 7.7 Hz, 2H), 1.52 – 1.41 (m, 6H), 0.98 (t, J = 6.9 Hz, 3H). GCP (CB): Mn = 21.4 kg mol^{-1}, Mw = 31.9 kg mol^{-1}, PDI = 1.5.

3. Nuclear Magnetic Spectroscopy (NMR)

^{1}H NMR spectroscopy was carried out on a Bruker AV400 or Bruker AVIII400 spectrometer. Samples were dissolved in 0.4 – 0.6 mL. High temperature NMR spectra were obtained via heating the samples in the NMR spectrometer at 363 K (90 °C).

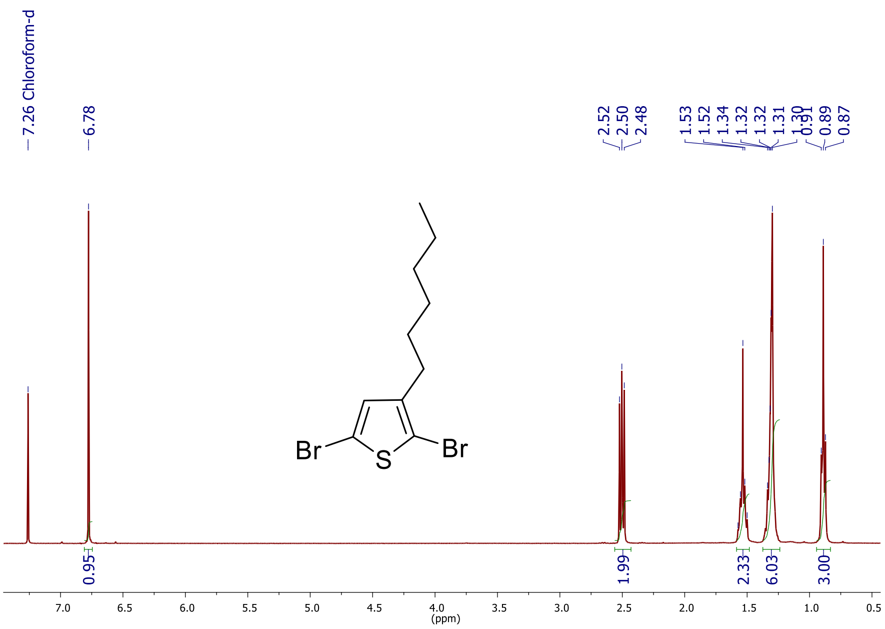

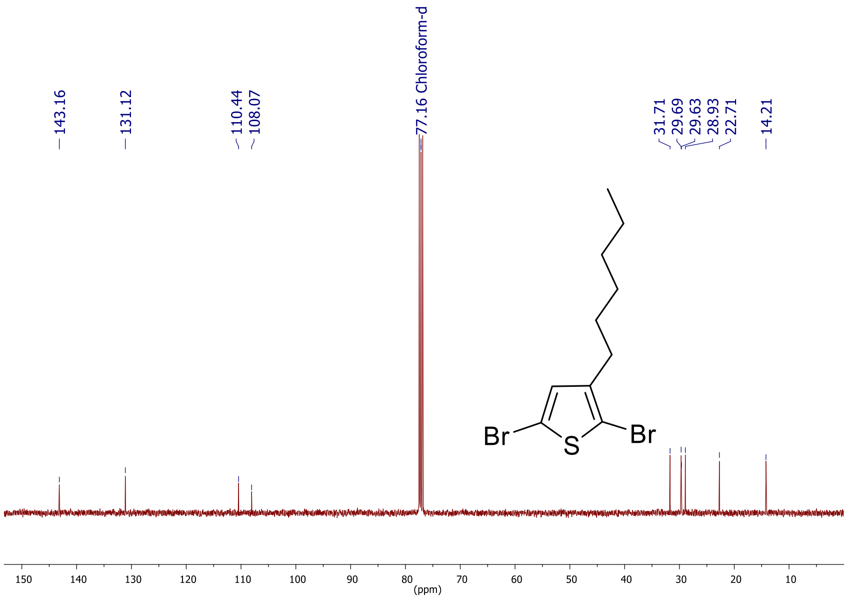

Figure S1. ^{1}H NMR (top) and ^{13}C NMR (bottom) of 2,5-dibromo-3-hexylthiophene (**1**) in $CDCl_3$. NMRs were compared to previous literature.[1]

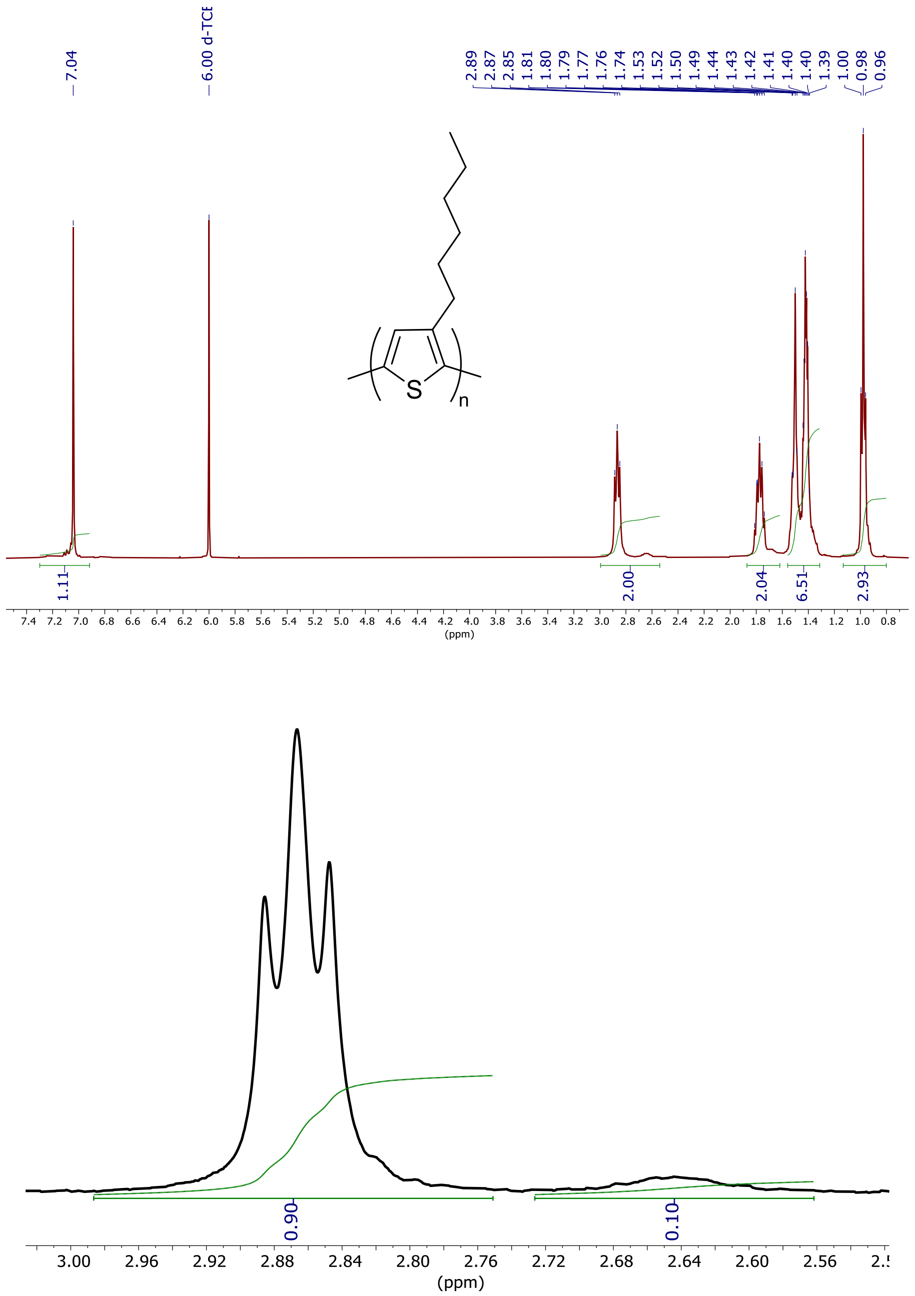

Figure S2. ^{1}H NMR of P3HT in d_2-TCE (top). Zoomed in of the triplet at 2.87 ppm and broad peak at 2.65 ppm are from the alpha protons on the alkyl chain. The integrated ratio between these peaks indicates the head to tail content/regioregularity.[2]

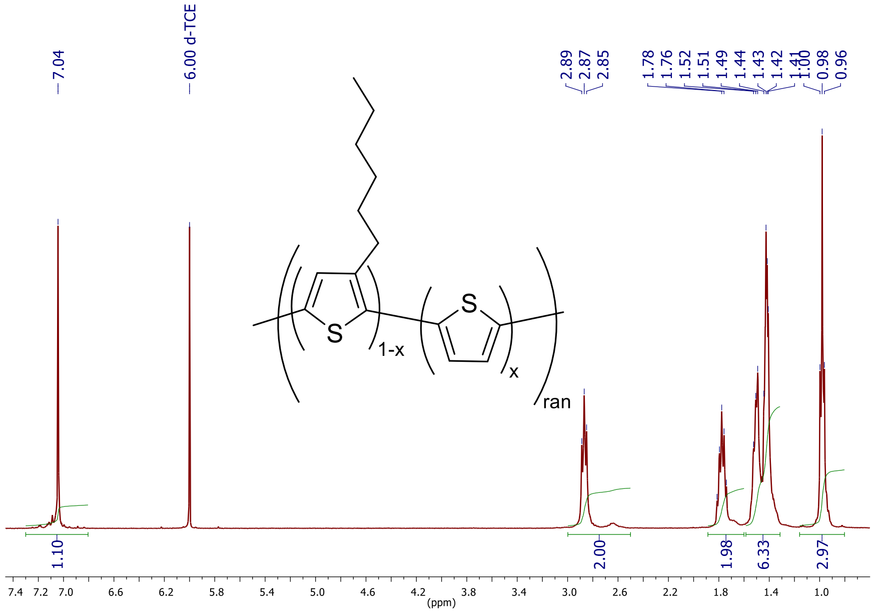

Figure S3. ^{1}H NMR of T_{ref} in d_2-TCE at 363 K (90 °C). X << 0.01

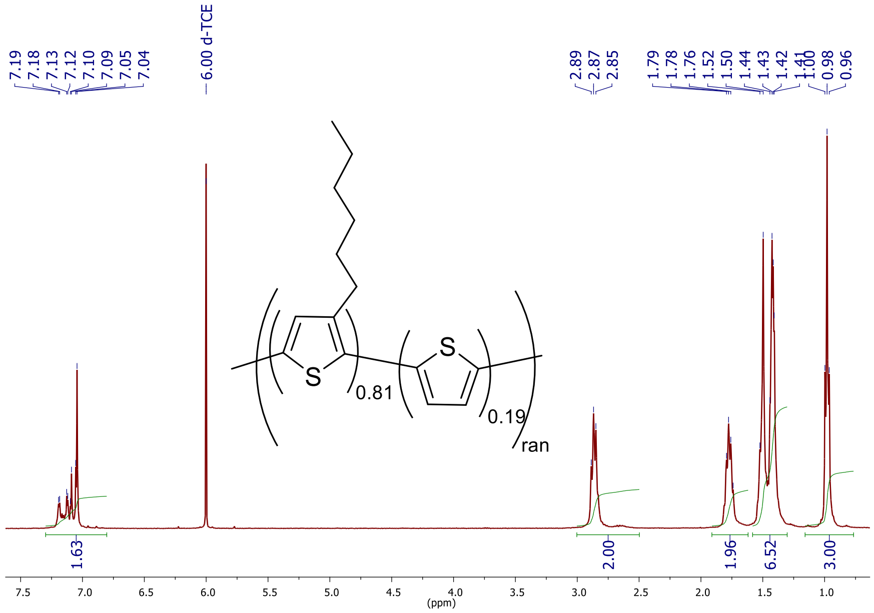

Figure S4. ^{1}H NMR of T24 in d_2-TCE at 363 K (90 °C).

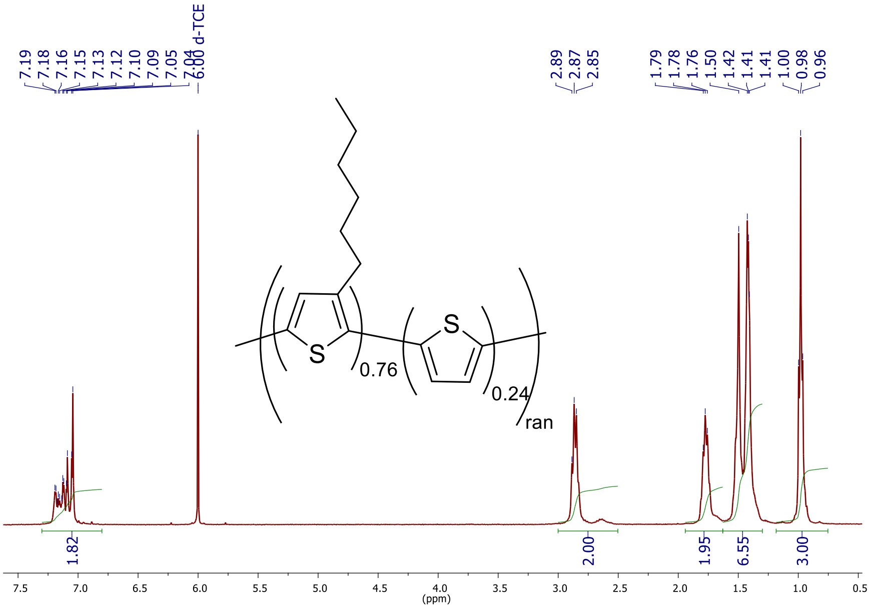

Figure S5. ^{1}H NMR of T24 in d_2-TCE at 363 K (90 °C).

Due to relaxation times commonly seen in conjugated polymer NMR, we note that the integration between the aromatic and aliphatic protons to not match in the P3HT spectra. Therefore, we have corrected the integration of 1.11 to 1.00 for P3HT and similarly for the other polymers in the series. If we then consider the statistical copolymer to have 1-x hexylthiophene units and x unsubstituted thiophene units, then we would expect 1+x aromatic protons and 2-2x aliphatic protons in the 2.8 ppm region (methylene group next to thiophene). With R defined as the ratio of aromatic protons to aliphatic protons (2.8 ppm region), we get R = (1+x)/(2-2x). Solving for x, we get x = (2R-1)/(2R+1). Thus, we calculate the thiophene content (x) from the ratio (R) of aromatic protons to aliphatic protons (2.8 ppm region) in the table below.

Table S1. Table summarising the values calculated to obtain the thiophene content.

Polymer	Aromatic integration	Corrected aromatic integration	R	x	Thiophene Content (%)
**P3HT**	1.11	1.00	0.50	0	0
**T_{ref}**	1.10	0.99	0.50	0	0
**T19**	1.63	1.47	0.73	0.19	19
**T24**	1.82	1.64	0.82	0.24	24

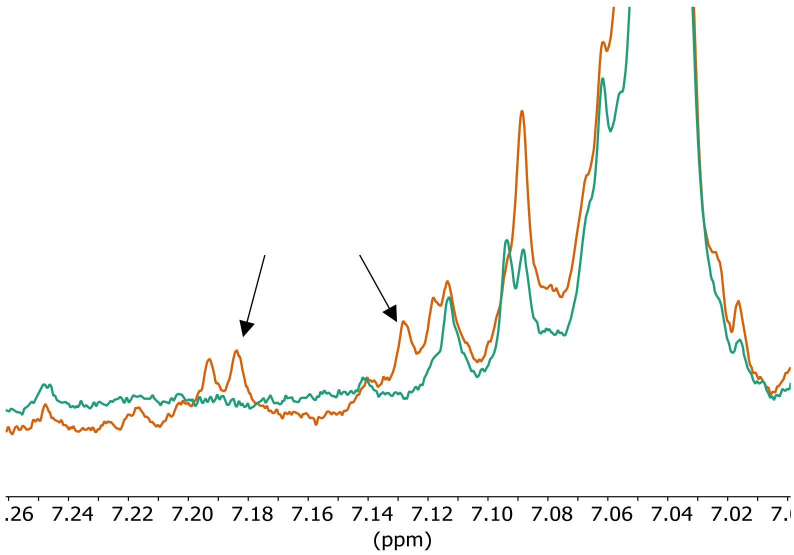

Figure S6. High temperature ^{1}H NMR spectra of showing the aromatic region of P3HT (green) and T_{ref} (orange). The arrows indicate the peaks observed in the T_{ref} spectra but not in the P3HT spectra.

4. Gel Permeation Chromatography (GPC)

GPC was carried out using Shimadzu Prominence GPC system, comprised of a SIL-20A auto sampler, LC-20AT liquid chromatograph, CTO-20A column oven, SPD-20A UV-Vis detector. HPLC grade chloroform was purchased from Acros Organics and polystyrene standards were purchased from Agilent. GPC Samples were prepared via dissolving the polymers in chlorobenzene at 1 mg mL^{-1} at 80 °C for 1 hr, then cooling to room temperature and filtering through a 0.45 μm PTFE filter. 100 μL was injected into the system and run at 1 mL min^{-1} in chlorobenzene through the oven at 80 °C. Analysis was carried out on Shimadzu's LabSolutions software.

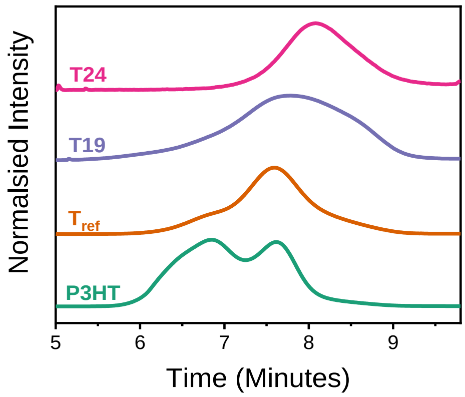

Figure S7. GPC traces of P3HT, T_{ref}, T19, T24. The peak for P3HT is bimodal indicating two different regimes however the molecule weight was estimated via integration over both peaks.

Table S2. Molar mass distributions and degree of polymerisation of P3HT, T_{ref}, T19, T24.

Polymer	M_w (kg mol^{-1})^a	M_n (kg mol^{-1})^a	Đ^b	Degree of Polymerisation^c
**P3HT**	52.8	39.7	1.3	239
**T_{ref}**	34.5	23.2	1.5	142
**T19**	46.8	26.1	1.8	179
**T24**	31.9	21.4	1.5	152

[a]Measured from the integration under the GPC trace vs polystyrene standards. [b]Đ = M_W/M_n. [c]Calculated using DP = $(f_T M_T + f_{3HT} M_{3HT})/M_n$ where f_T and f_{3HT} represent the fraction of thiophene and 3-hexylthiophene monomer calculated from NMR integrations and M_T and M_{3HT} represent the molecular weight of the monomers.

5. Thermal characteristics

TGA was carried out on a TA instruments Q500 using a platinum pan at 10 °C min^{-1} between 25 - 800 °C under nitrogen then 800 – 1000 °C under air. Between 0.5 – 3 mg of polymer powder was used for TGA. DSC was carried out on TA Instruments DSC25 running at 10 °C min^{-1} under a nitrogen atmosphere. Between 1 – 5 mg of polymer powder was used for DSC.

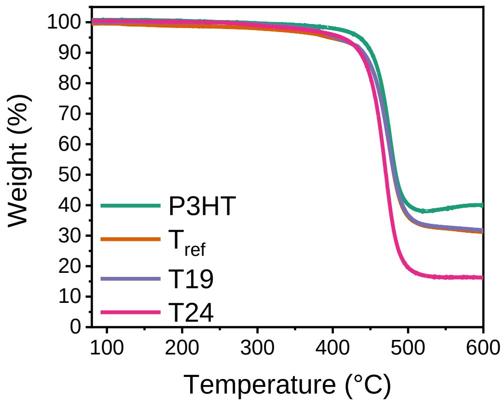

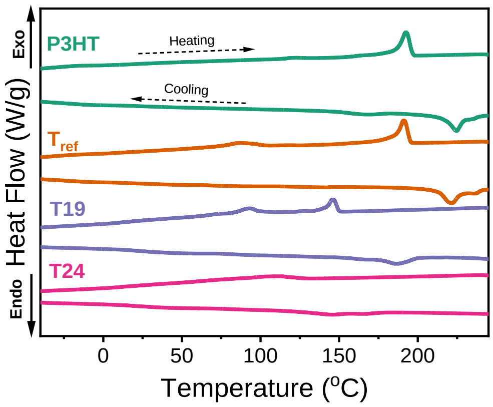

Figure S8. TGA (left) and DSC (right) traces of P3HT, T_{ref}, T19, T24. The second cycle is shown for the DSC traces.

Table S3. Table showing the thermal properties estimated from TGA and DSC spectra of P3HT, T_{ref}, T19, T24.

Polymer	Onset of Degradation (°C)	T_c (°C)	T_m (°C)
**P3HT**	456	192	226
**T_{ref}**	448	191	221
**T19**	446	146	187
**T24**	446	110	146

6. Thin film fabrication

Non-aligned polymer thin films were fabricated by spin coating thin films onto glass sides from 10 mg mL^{-1} polymer solutions in ODCB dissolved at 80 °C. Solutions were spun at 2000 rpm for 90 seconds then 8000 rpm for 30 seconds from 80 °C solutions in air on a Laurell Technologies Corporation Model WS-650MZ-23NPPB spin coater. Glass slides were cleaned by sonicating in soapy water, DI water, acetone, and IPA for 15 minutes. Prior to spin coating substrates were plasma cleaned using a Harrick Plasma PDC-32G plasma cleaner for 30 minutes. Note that decreasing the side chains density decreases the polymer solubility in nonpolar and apolar solvents. One should consider low concentration (<20 mg mL^{-1}) to ensure complete solubility of the polymer before casting.

The aligned polymer films were prepared by doctor-blading a hot solution in ODCB (10 mg mL^{-1}) at 160 °C on cleaned glass slides covered with a sacrificial polymer film of water-soluble NaPSS (10 mg mL^{-1} aq). The orientation of the films by high-temperature rubbing followed the protocol described in previous publications.[3] [4] Rubbing is performed by using a homemade set-up. It consists of a rotating cylinder covered with a polyester cloth and a translating hot plate.

7. UV-Vis spectroscopy

Solution and thin film UV-Vis spectroscopy of the non-aligned films neutral films was carried out on a Shimadzu UV3600 UV-Vis-NIR spectrometer. Temperature dependent solution UV-Vis spectroscopy was carried out in quartz cuvettes from solutions of polymers in chlorobenzene (CB) at 0.01 mg mL^{-1} at 20 to 100 °C using a VICI DBS PCB 1500 Plus Peltier Cryobath with cuvette holder as the heater/cooler. All spectra were baselined to chlorobenzene at 20 °C.

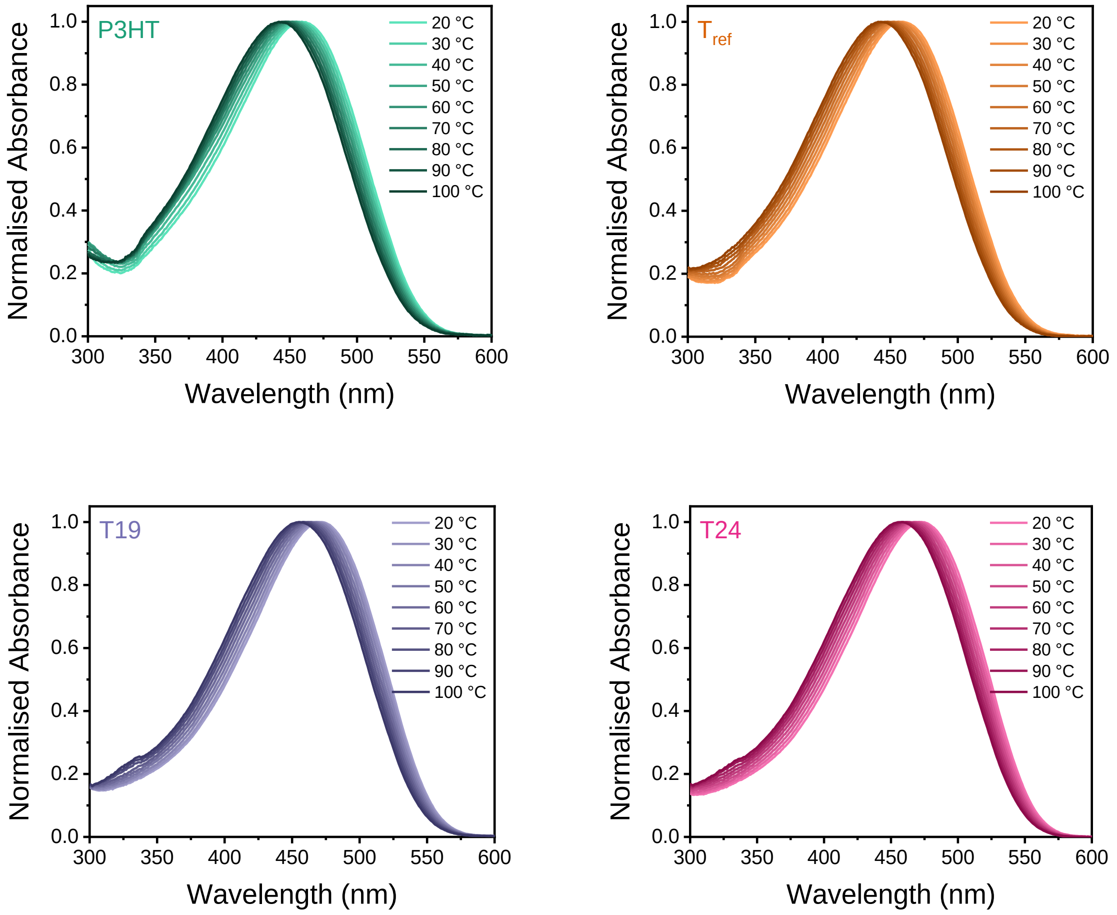

Figure S9. Temperature dependent solution UV-Vis spectra of P3HT, T_{ref}, T19, T24 in chlorobenzene at 0.01 mg mL^{-1} from 20 – 100 °C. The spectra are normalised to the lambda max.

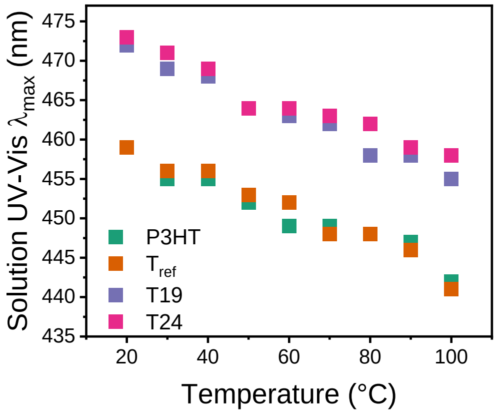

Figure S10. Plot of λ_{max} obtained from solution UV-Vis spectra of P3HT, T_{ref}, T19, T24 vs temperature.

The orientation of the aligned polymer films was probed by UV–Vis–NIR absorption (350 – 2500 nm) using a Varian Cary5000 spectrometer with polarized incident light (spectral resolution of 1 nm).

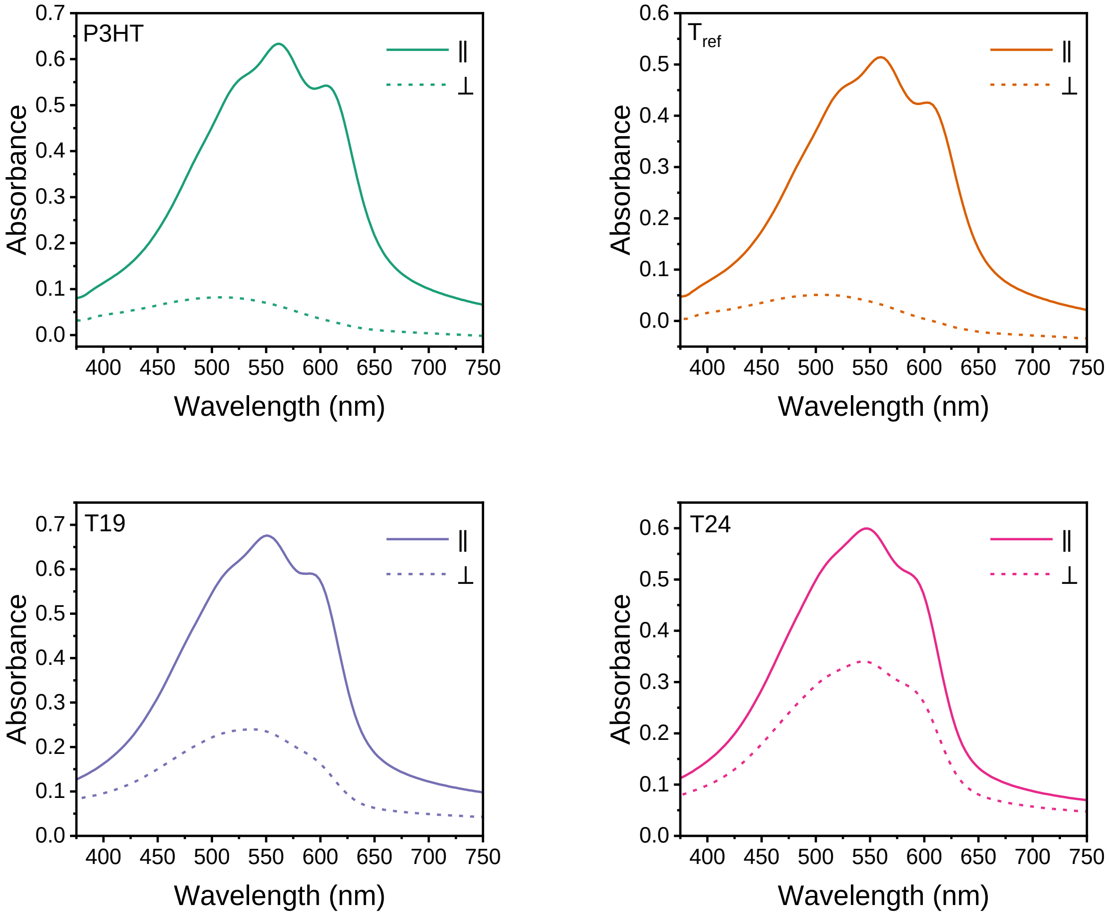

Figure S11. UV-Vis spectra of P3HT, T_{ref}, T19, T24 aligned polymer films in the parallel (solid line) and perpendicular (dotted line) direction to the rubbing direction.

8. Electrochemistry and Spectroelectrochemical Experiments

CV was carried out using a PalmSens EmStat3 potentiostat with degassed acetonitrile with 0.1 M tetrabutylammonium hexafluorophosphate as the supporting electrolyte measured at 50 mV s^{-1} scan rate. The reference, counter and working electrode were Ag/Ag^{+}, platinum and glassy carbon, respectively. Thin films of each polymer were drop cast from 1 mg mL^{-1} chloroform solutions onto glassy carbon electrode. Ferrocene was used as the standard, measured using 0.01 M ferrocene in degassed acetonitrile with 0.1 M tetrabutylammonium hexafluorophosphate as supporting electrolyte at a scan rate of 50, 100 and 200 mV s^{-1}. Diameter of the working electrode is 3 mm.

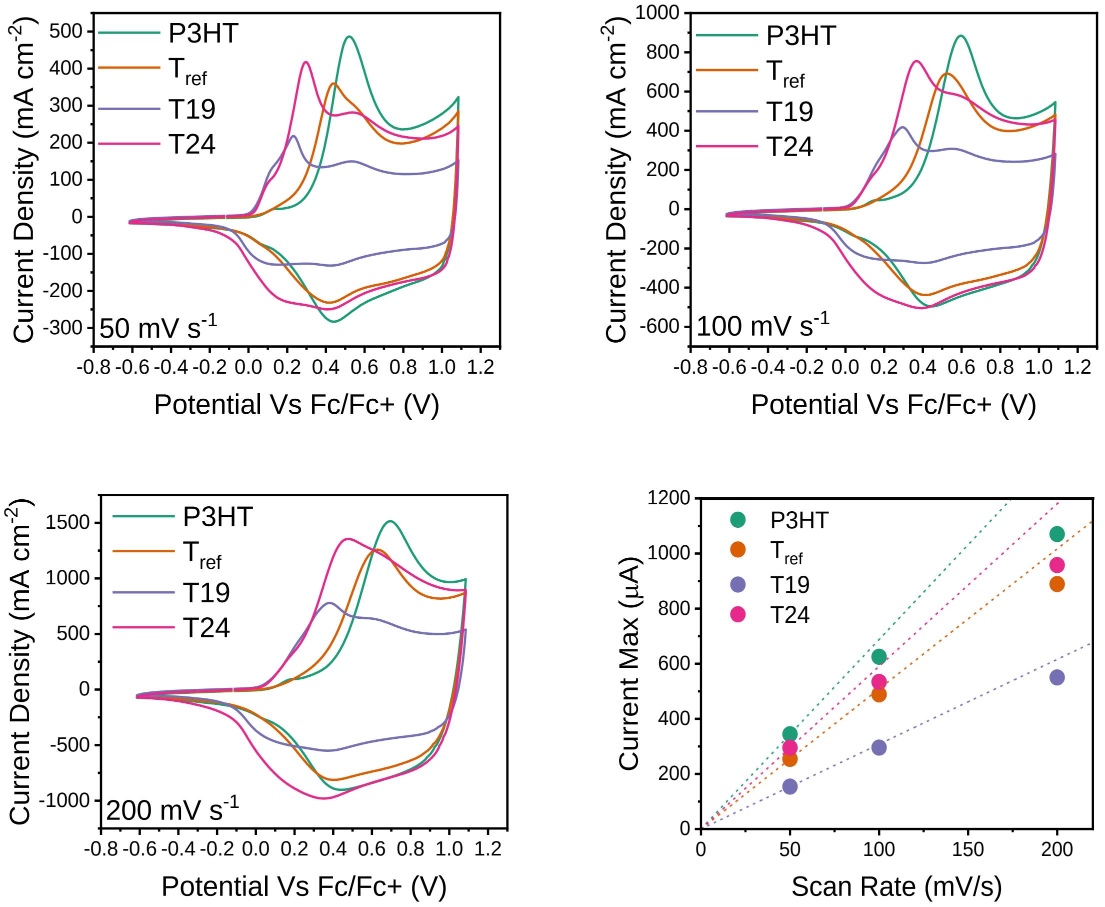

Figure S12. Cyclic voltammograms of P3HT, T_{ref}, T19, T24 at 50, 100 and 200 mV s^{-1} (top left, top right and bottom left respectively). Plot of current max vs scan rate across the polymer series (bottom right). The dotted line of each colour represents the linear extrapolation from 0 to 50 mV s^{-1}.

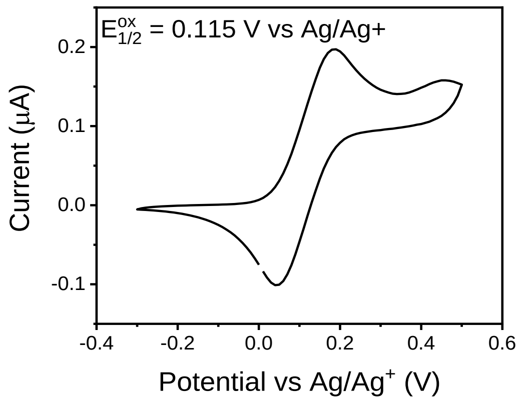

Figure S13. Voltamagram of 0.01 M ferrocene in acetonitrile vs Ag/Ag^+ used to calibrate to voltamegrams.

For the spectroelectrochemistry experiments, thin films of P3HT and T24 were spun onto 2 x 2 cm ITO coated glass at 2000 rpm for 60 seconds then 8000 rpm for 30 seconds from 10 mg mL^{-1} ODCB solutions at 80 °C. The polymer films were then swabbed down to 1 x 2 cm using IPA and cotton buds, then the slides were scored and split in half to produce 1 x 1 cm polymer

films on 1 x 2 cm slides. The exposed ITO on the substrate was clipped using crocodile clips and submerged into degassed acetonitrile with 0.1 M tetrabutylammonium hexafluorophosphate as the supporting electrolytes in a quartz cuvette. Platinum wire, Ag/Ag^{+} and the ITO coated glass slide were used as the counter, reference and working electrode respectively. ITO slides were cleaned using, soapy water, DI water, acetone and IPA via sonicating for 15 minutes. The whole cuvette was placed into the Shimadzu UV3600 UV-Vis-NIR spectrometer and potentials were applied using PalmSens EmStat3 potentiostat. Spectra were recorded after 1 minute of applying each new potential.

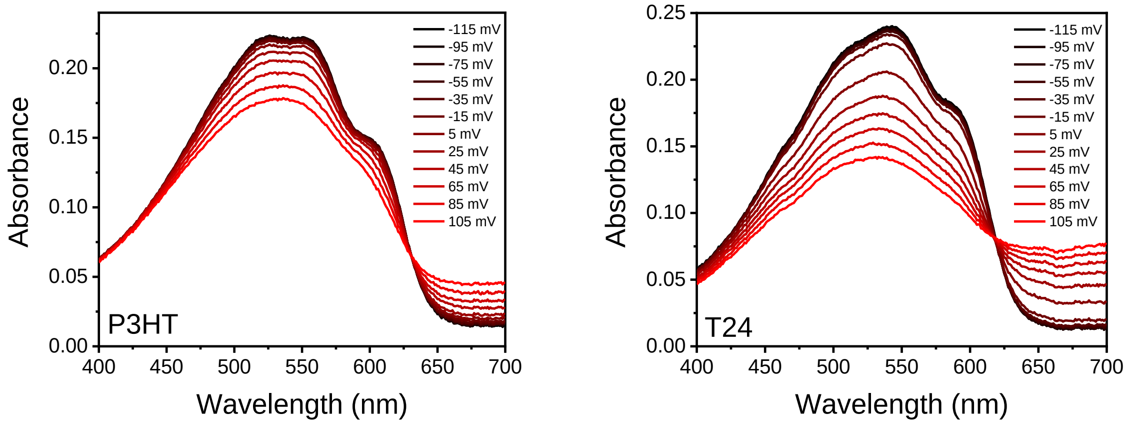

Figure S14. UV-Vis spectra of P3HT (left) and T24 (right) at increasing potential vs Fc/Fc^{+}.

9. Work Function Measurements

Photo-Electron Spectroscopy in Air (PESA) has been performed on an AC-2 Model from Riken Instruments. UV photons are emitted from a deuterium lamp, then monochromated by a grating spectrometer and finally focused on the sample film. The photoelectrons emitted by the sample are detected by an open counter. When the sample's surface is bombarded with a slowly increasing amount of UV energy, photoelectrons start to emit at a certain energy level which corresponds to the photoelectron work function. Thin films were fabricated using 10 mg ml^{-1} ODCB solutions, doctor bladed onto ITO coated glass substrates.

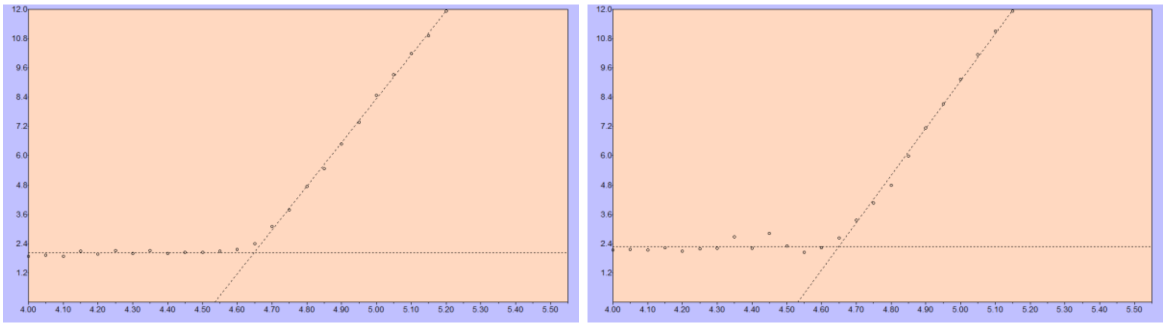

Figure S15. Photoemission spectra of P3HT thin films with increasing energy (eV) along the x-axis against standardised photoelectron yield (Yield^n, where n is 0.33) along the y-axis.

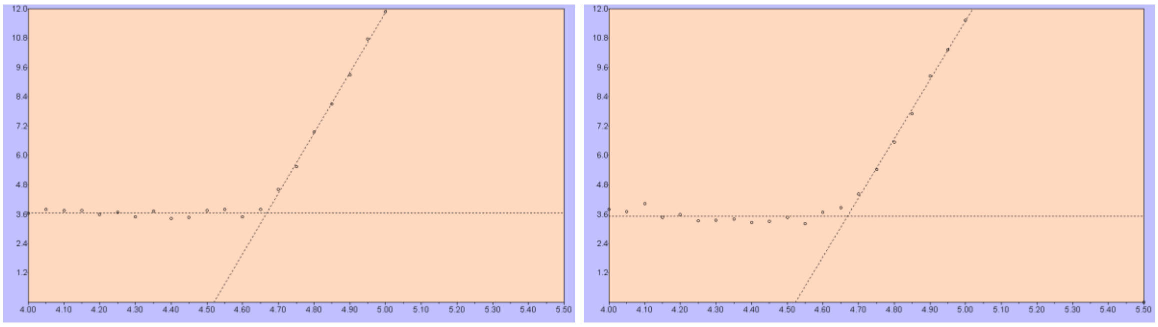

Figure S16. Photoemission spectra of T_{ref} thin films with increasing energy (eV) along the x-axis against standardised photoelectron yield (Yield^n, where n is 0.33) along the y-axis.

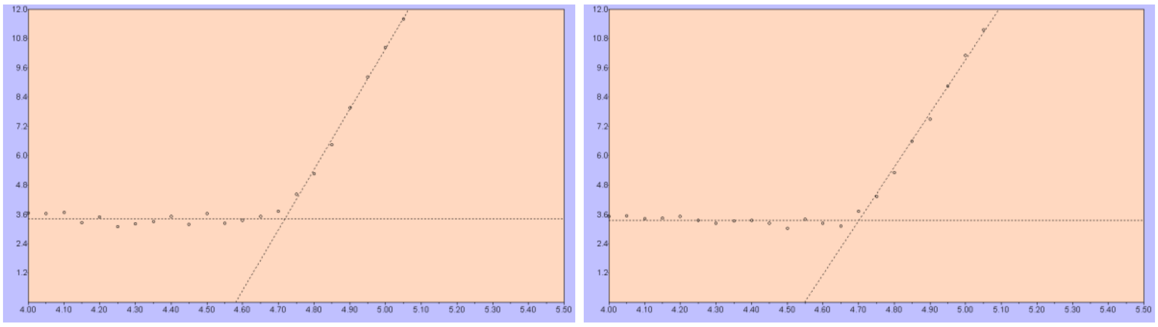

Figure S17. Photoemission spectra of T19 thin films with increasing energy (eV) along the x-axis against standardised photoelectron yield (Yield^n, where n is 0.33) along the y-axis.

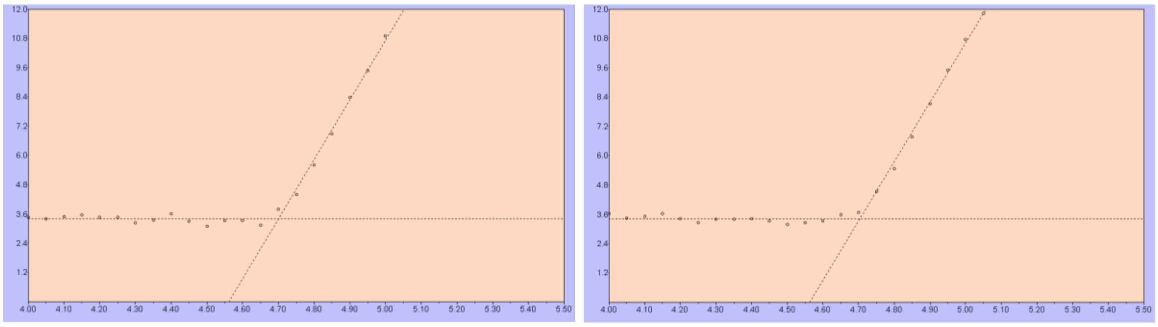

Figure S18. Photoemission spectra of T24 thin films with increasing energy (eV) along the x-axis against standardised photoelectron yield (Yield^n, where n is 0.33) along the y-axis.

Table S4 Work functions of P3HT, T_{ref}, T19, T24.

**Polymer**	**Work Function (eV)**[a]	
	**1**	**2**
**P3HT**	4.65	4.65
**T_{ref}**	4.67	4.67
**T19**	4.72	4.70
**T24**	4.70	4.70

[a]Obtained from thin films on ITO coated glass substrates.

10. X-ray diffraction

Grazing incidence x-ray diffraction measurements in the out-of-plane direction were carried out on PANalytical X'Pert Pro diffractometer using Cu K_α X-rays, setup in grazing incidence configuration. Thin films were drop cast onto boron doped single side polished silicon substrates of <100> orientation, purchased form PI-KEM, from 20 mg mL^{-1} polymer solutions in ODCB at 80 °C. Films were covered with glass petri dish and allowed to dry overnight in air. Due to the low power of the diffraction measurement, thick films were required to record significant signals for analysis. Sufficient thickness was not achieved by spin-coating, so the polymers were drop casted instead. Therefore, we note here that the morphology-property relationships drawn for non-aligned films rely on the assumption that the order observed for drop-casted films is representative for the spin-coated films. The films were doped via immersion in 2 mg mL^{-1} F4TCNQ solutions in degassed acetonitrile overnight in air on a shaker plate. The films were then washed with acetonitrile and dried under vacuum. We would like to note here that using the Scherrer equation limits the analysis as beak broadening is due to paracrystalline disorder, not finite grain size which is assumed by the Scherrer equation. These results are shown in lieu of a more complete linewidth analysis.[5]

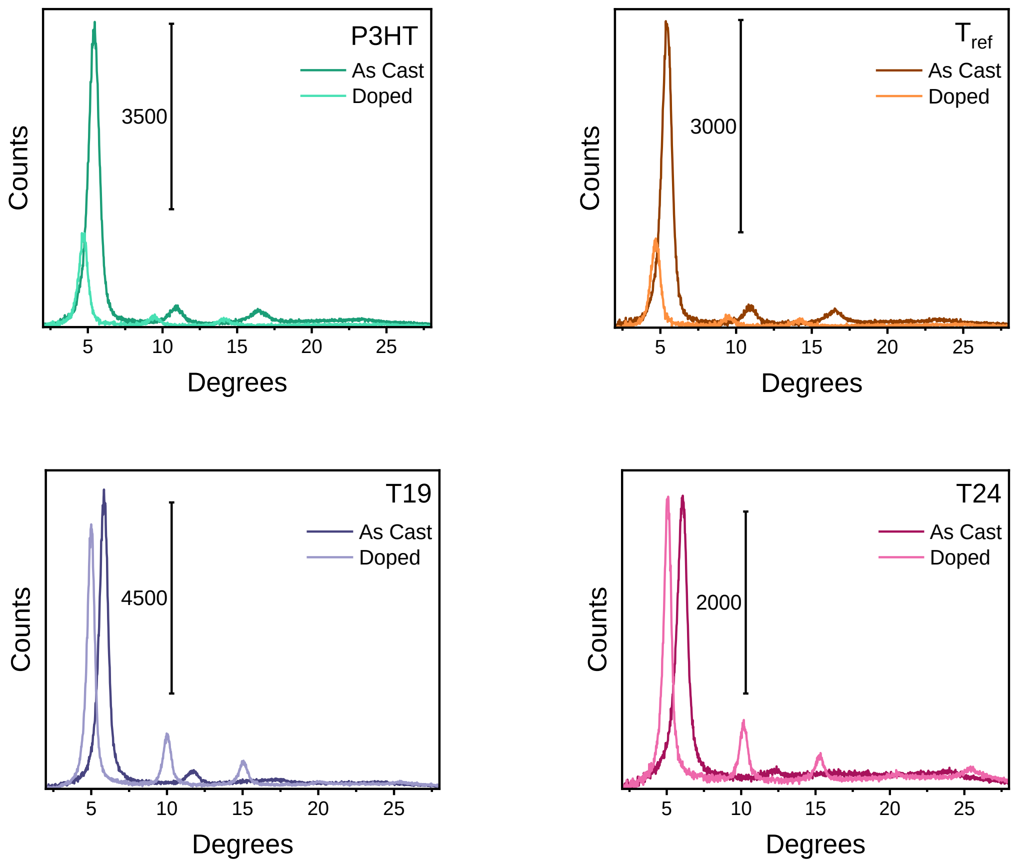

Figure S19. GIXRD diffraction patterns in the out-of-plane direction of P3HT, T_{ref}, T19, T24 drop cast films from 20 mg mL^{-1} ODCB solutions at 80 °C in the pristine and doped state. The scale bar represents counts.

Table S5. Table showing the d-spacings, FWHM and coherence lengths of the neutral and doped non-aligned drop cast films.

Polymer	Neutral			Doped		
	d(100) (Å)[a]	FWHM[b]	Coherence Lengths (Å)	d(100) (Å)[a]	FWHM[b]	Coherence Lengths (Å)
**P3HT**	16.3	0.90	92	18.8	0.76	109
**T_{ref}**	16.3	0.82	101	18.9	0.76	109
**T19**	15.2	0.73	113	17.7	0.62	136
**T24**	14.6	0.91	92	17.5	0.64	130

[a]Calculated using Braggs law using Cu Kα (1.5406 Å) as the wavelength. [b]FWHM was obtained from fitting a gaussian peak to the (100) peak using Origin Pro's fitting tool. [c]The coherence lengths were calculated using the Scherrer equation.

11. Roughness measurements

AFM was carried out in air using a Bruker Dimension Icon system. ScanAsyst Air tips were used to image the samples in PeakForce Quantitative Nanomechanical Property Mapping (QNM) mode. Roughness was calculated using Nanoscope Analysis software.

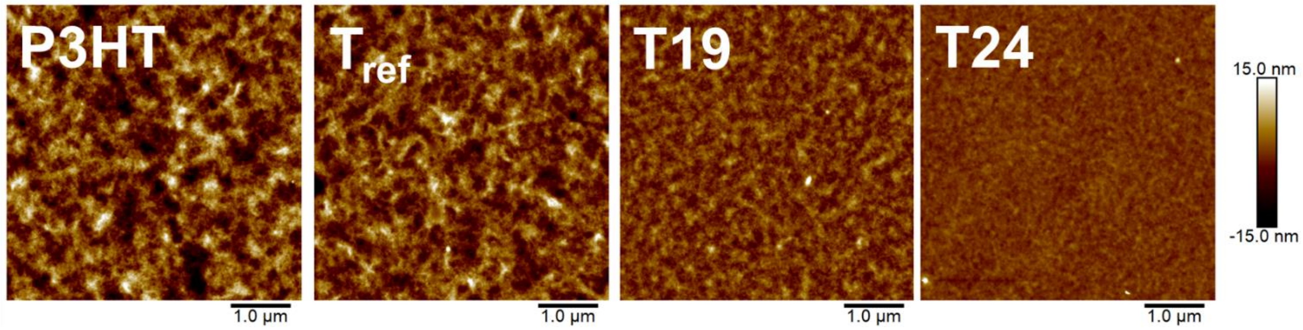

Figure S20. AFM images of P3HT, T_{ref}, T19, T24.

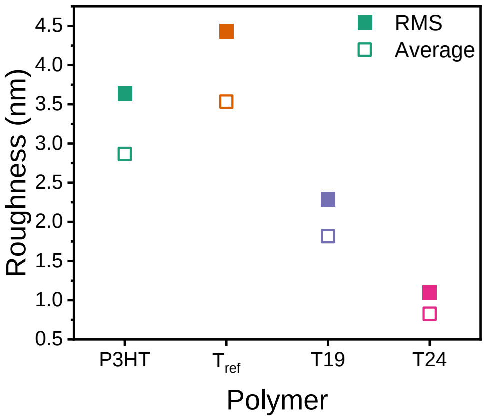

Figure S21. Plot of route mean square (RMS) (closed squares) and average (open squares) roughness of P3HT, T_{ref}, T19, T24 obtained from AFM images.

12. Organic Field Effect Transistor (OFET) Measurements

The back-gate and gate dielectrics were chosen to be highly n-doped silicon and thermally grown SiO_2 (300 nm), respectively. After cleaning Si/SiO_2 substrates with oxygen plasma at 300W for 10 min, the substrates were then immersed in 3 wt% PTS/toluene solution for 15 hours at 90 °C. The excess PTS on Si/SiO_2 substrates were cleaned by sonication with toluene, followed by toluene, acetone and isopropanol rinse. The polymers solutions were pre-heated at 80°C for 1-2 hours before film deposition. The polymers (10 mg mL^{-1}) in ODCB were spin-coated on PTS-functionalized Si wafer. The Cr/Au electrodes (5/250 nm) were thermally evaporated under high vacuum (10^{-6} mbar) as the source and drain electrodes (W/L = 1000/20). The prepared OFETs were placed in a nitrogen glove box prior to testing. The mobility was calculated from the gradient of the transfer curve after any ‘kinks’ in the curve.[6]

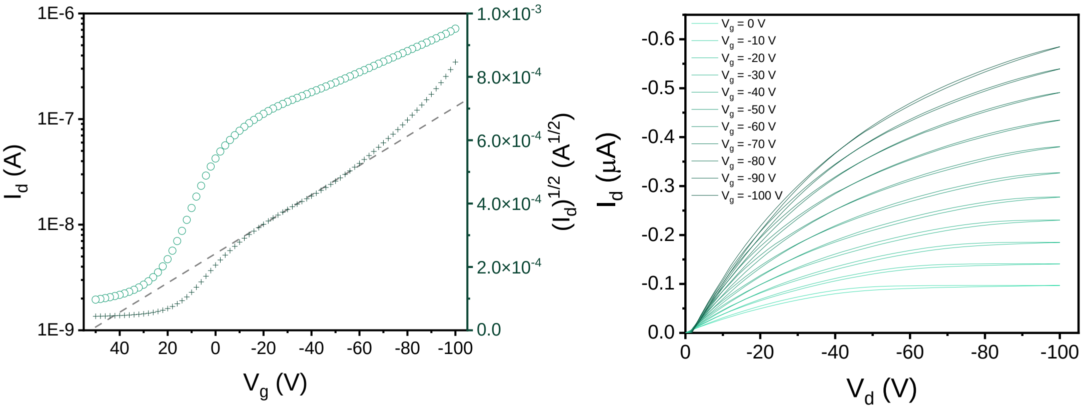

Figure S22. Transfer characteristics at a drain voltage of -60 V (left) and Output curve (right) of a P3HT OFET. The saturation mobility was extracted from the gradient of the grey line on the $(I_d)^{1/2}$ vs V_g plot.

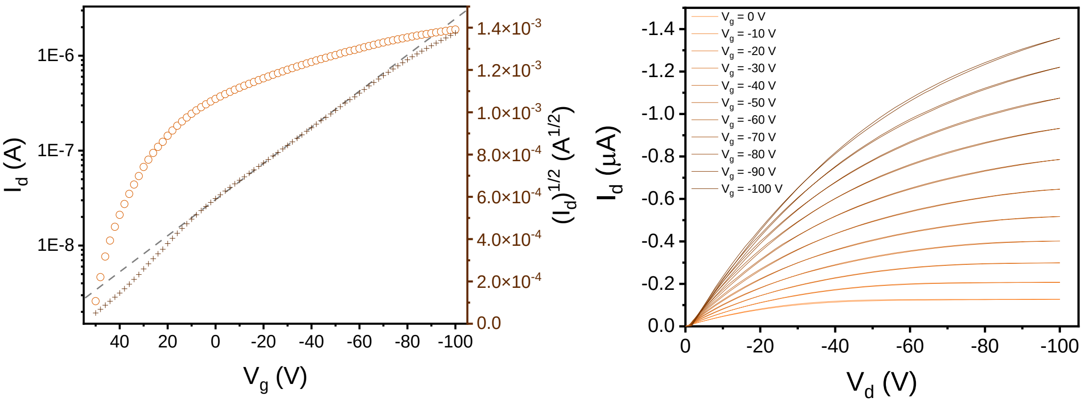

Figure S23. Transfer characteristics at a drain voltage of -60 V (left) and Output curve (right) of a T_{ref} OFET. The saturation mobility was extracted from the gradient of the grey line on the $(I_d)^{1/2}$ vs V_g plot.

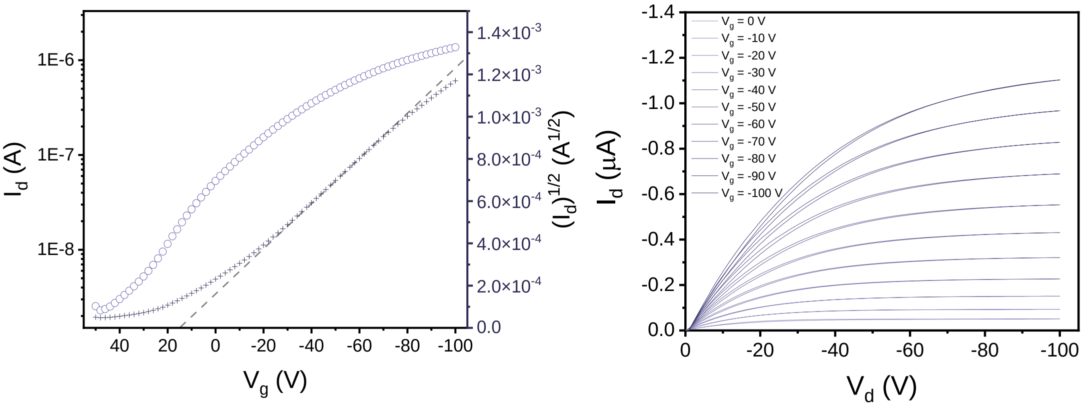

Figure S24. Transfer characteristics at a drain voltage of -60 V (left) and Output curve (right) of a T19 OFET. The saturation mobility was extracted from the gradient of the grey line on the $(I_d)^{1/2}$ vs V_g plot.

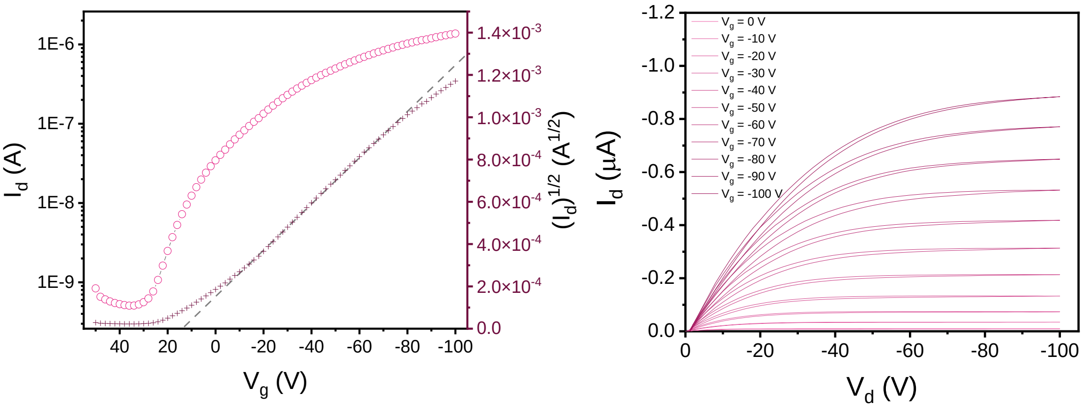

Figure S25. Transfer characteristics at a drain voltage of -60 V (left) and Output curve (right) of a T24 OFET. The saturation mobility was extracted from the gradient of the grey line on the $(I_d)^{1/2}$ vs V_g plot.

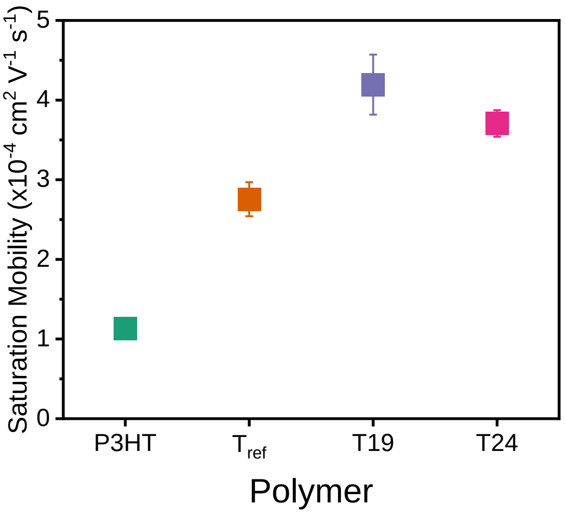

Figure S26. Plot of the average saturation hole mobility measured from 3 OFET devices of P3HT, T_{ref}, T19, T24. The OFETs were in bottom-gate, top-contact configuration and SiO_2 as the dielectric layer, along with PTS as a self-assembled monolayer.

Table S6. Saturation mobility of P3HT, T_{ref}, T19, T24 thin films.

**Polymer**	**Saturation Mobility ($x10^{-4}$ cm^2 V^{-1} s^{-1})**
**P3HT**	1.1 ± 0.08
**T_{ref}**	2.8 ± 0.21
**T19**	4.2 ± 0.38
**T24**	3.7 ± 0.17

13. Conductivity, UV-Vis Absorbance and FTIR Spectroscopy of Doped Non-Aligned Thin Films

Glass substrates patterned with van der Pauw contacts (1 mm contacts at each corner) was chosen to permit conductivity, UV-Vis spectroscopy, and FTIR measurements on a single sample. Substrates were first prepatterned with Cr/Au electrodes (5/25 nm) by thermal evaporation through a shadow mask, then cleaned by sequential sonication in Decon 90, DI water, acetone, and isopropanol, dried under nitrogen flow, and exposed to oxygen plasma for 10 minutes. After transferring to a nitrogen glovebox (MBraun Labmaster 130, <1 ppm H_20, O_2), polymer thin films were spun cast at 1500 rpm for 60 secs (Specialty Coating Systems G3P) from 10 mg mL^{-1} ODCB solutions at 80 °C, then annealed at 180 °C for 20 mins under N_2. Thin films were doped with two different concentrations under nitrogen atmosphere. HighSqP doping was carried out by depositing a 1 mg mL^{-1} solution of F4TCNQ in MeCN, leaving on the films for 60 seconds, then removed via spinning at 8000 rpm for 30 seconds. This was followed by another 1 mg mL^{-1} solution of F4TCNQ in ODCB leaving on the film for 120 seconds, then removed by spinning at 8000 rpm for 30 seconds. LowSqP doping was carried out via depositing a 0.1 mg mL^{-1} solution of F4TCNQ in MeCN, leaving on for 10 secs, then spinning off at 8000 rpm for 30 secs. Film thickness was measured using a Bruker DekTak XT. The higher performing batch of P3HT used to confirm our findings (named here P3HT99) was synthesised previously.[7] The RR% was measured to be above 99 % measured by high temperature NMR. The M_n, M_w and Đ are 49 kDa, 75 kDa and 1.5 respectively.

Conductivity measurements were measured on a Karl Suss probe station under a nitrogen atmosphere (<20 ppm O_2) using an Agilent 4155B sourcemeter following the standard van der Pauw method. Four I-V measurements of each sample were performed, corresponding to sourcing current between each pair of adjacent contacts, while measuring the voltage at the other pair of contacts. These data were checked for current reversal and reciprocity consistency to <5% variance, following NIST recommendations; the van der Pauw equation was then used to determine the sheet conductivity.

Table S7. Table showing the conductivity results of the non-aligned polymer thin films.

Polymer	Conductivity (S cm^{-1})		Thickness (nm)[c]	
	High[a]	Low[b]	High[a]	Low[b]
**P3HT**	3.1	0.3	40	38
**T_{ref}**	7.3	1.3	32	30
**T19**	14.2	3.2	35	34
**T24**	19.3	4.6	36	35
**P3HT99**	13.9	4.6	26	20

[a]HighSqP F4TCNQ doping conditions. [b]LowSqP F4TCNQ doping conditions. [c]Average over four measurements.

UV-Vis measurements were performed on a Shimadzu UV-3600i UV-Vis-NIR spectrometer. IR spectroscopy was carried out on a Bruker Vertex 70v FT-IR spectrometer under vacuum using a DLaTGS detector. The data drops below 0 absorbance as the beam splitter is only rated to 6000 cm^{-1} in the spectrometer. Thin film interference present in the data were filtered out in MATLAB using a stopband filter. To extract the F4TCNQ anion concentration we used the same fitting parameters as our previous work. [1]

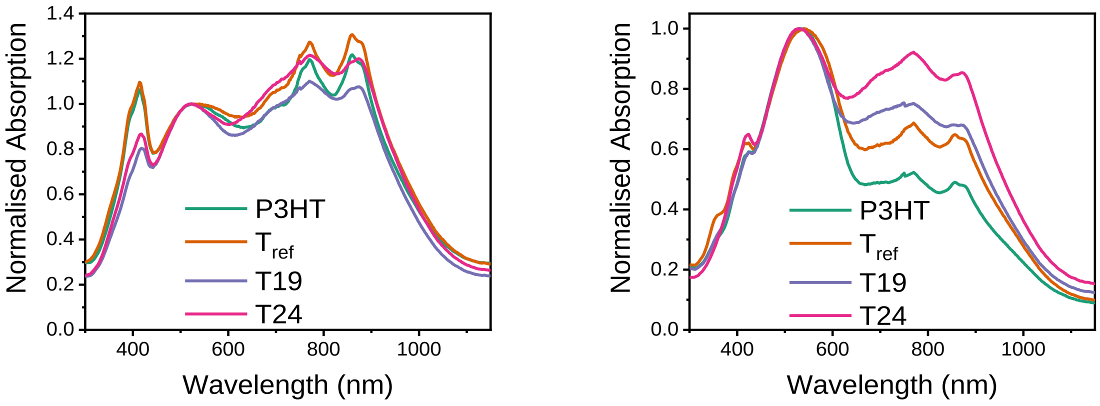

Figure S27. Thin film UV-Vis absorbance spectra of the polymer series doped under high (left) and low (right) doping conditions.

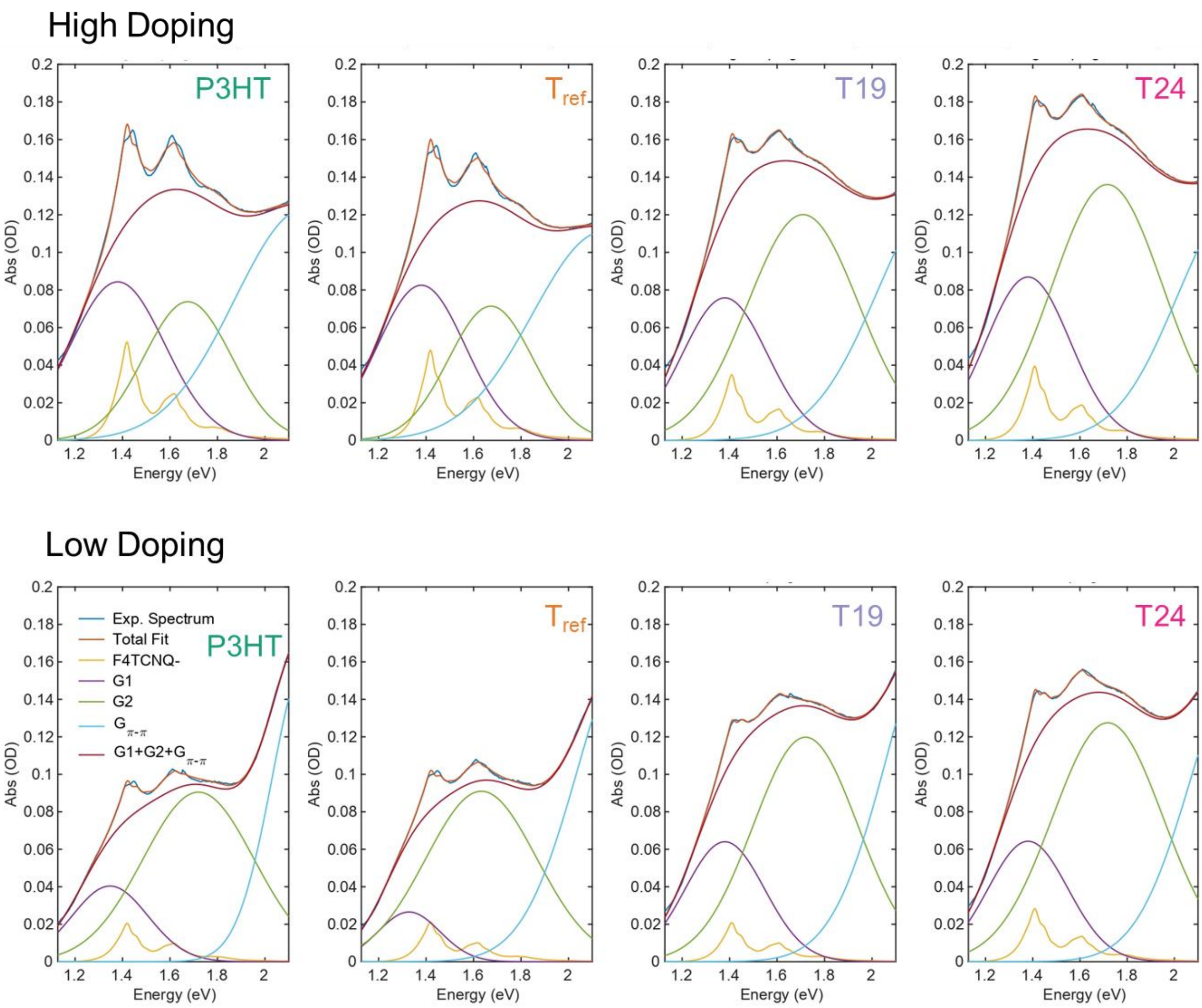

Figure S28. Fits of the high (top row) and low (bottom row) doped UV-Vis spectra using the same fitting parameters according to our previous work.[1]

Table S8. Table showing the extracted F4TCNQ concentrations and estimated doped mobility.

**Polymer**	**F4TCNQ Concentration (x10^{20} cm^{-3})**		**Doped Mobility (cm^2 V^{-1} s^{-1})**	
	**High[a]**	**Low[a]**	**High[b]**	**Low[b]**
**P3HT**	1.9 ± 0.23	0.8 ± 0.08	0.10	0.02
**T_{ref}**	2.3 ± 0.18	1.0 ± 0.09	0.20	0.09
**T19**	1.4 ± 0.14	0.9 ± 0.08	0.62	0.24
**T24**	1.6 ± 0.14	1.1 ± 0.09	0.76	0.25

[a]Extracted from the fitting results. [b]Estimated using the relation $\sigma = \mu e n$ where σ is the measured conductivity (S cm^{-1}), e is the electric charge (1.602 x 10^{-19} C), n is the F4TCNQ concentration (cm^{-1}) and μ is the doped mobility (cm^2 V^{-1} s^{-1}).

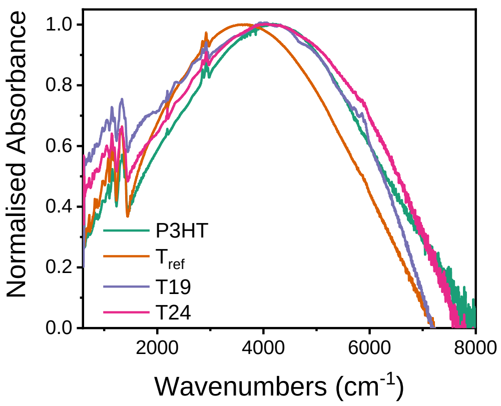

Figure S29. FTIR spectrum of low doped polymer films.

14. UV-Vis-NIR Spectroscopy, Electron Diffraction and Thermoelectric Characteristics of Doped Aligned Thin Films

The doping was performed following the incremental concentration doping (ICD) procedure introduced in a previous publication.[8 9, 10] Samples were immersed for 10 s in the dopant solution of increasing concentration. Such short doping times are sufficient to reach saturation of the film doping level as shown earlier for P3HT. No rinsing step was conducted as it results in de-doping of the films. Doping was performed in a jacomex glovebox under inert atmosphere (< 1 ppm O2 and < 1 ppm H2O).

All devices were fabricated on glass substrates cleaned by ultrasonication in acetone, ethanol, hellmanex and deionized water (x3 times). The cleaned substrates were dried under nitrogen and exposed to plasma prior to film deposition. Gold electrical contacts (40 nm thick) in a four-points probe geometry (1 mm spacing between electrodes, 5 mm length) were deposited by evaporation at an average rate of 4–6 Å s^{-1} through a shadow mask. The geometry of deposited gold electrodes was used to measure the charge transport and thermopower on a same sample in both parallel and perpendicular directions to the rubbing. Oriented polymer films were floated on water and carefully recovered on the device with pre-deposited gold electrodes. They were subsequently doped using the ICD protocol.[11] Four-point probe measurements of electrical conductivity were performed using a Keithley 2634B and a Lab Assistant Semiprobe station in a Jacomex glovebox under N2 atmosphere. The resistivity ρ was derived from the sheet resistance R such that ρ = 1.81.R.t where t is the film thickness (the geometrical correction factor was determined following the method in reference 3). The film thickness was extracted from the UV-Vis absorbance spectra using the calibration curves described below. The average conductivity value for a given rubbing temperature was taken as the average of two devices.

Thermopower measurements were conducted on the same devices. The thermopower was measured using a differential temperature method. A variable temperature gradient ΔT was established and the corresponding thermovoltage ΔV was measured. The Seebeck coefficient was extracted from the slope of ΔV versus ΔT. Calibration of the Seebeck coefficient measurement was performed using a constantan wire.

The orientation of the aligned polymer films was probed by UV–Vis–NIR absorption (350 – 2500 nm) using a Varian Cary5000 spectrometer with polarized incident light (spectral resolution of 1 nm).

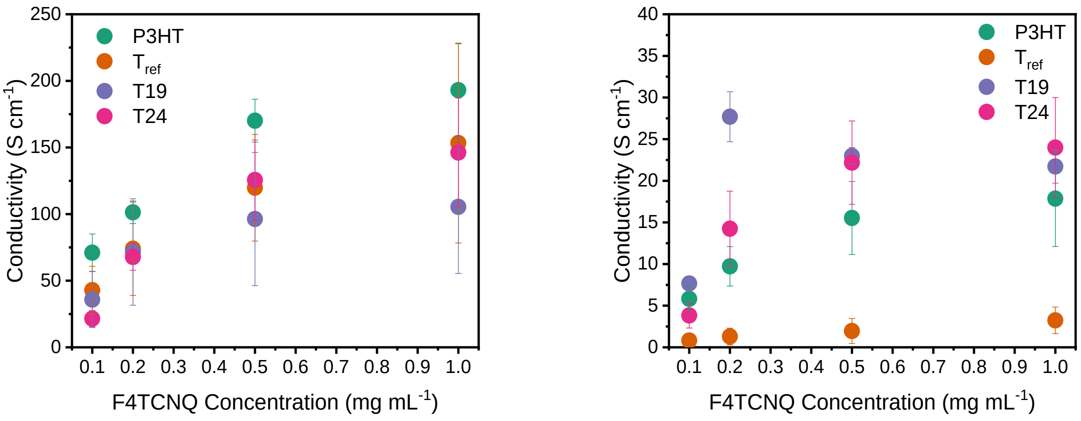

Figure S30. Measured conductivity of P3HT, T_{ref}, T19, T24 aligned thin films in the parallel (left) and perpendicular (right) direction to the rubbing direction. Error bars arise from the average of two measurements.

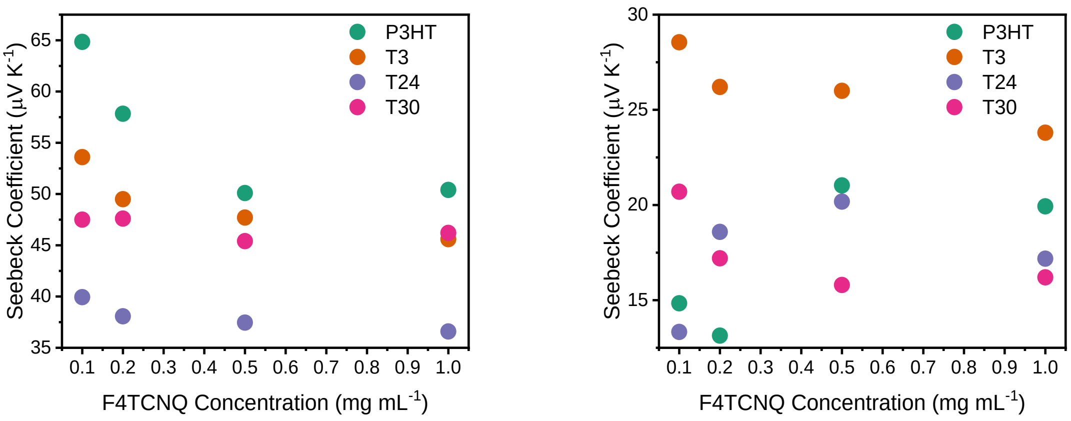

Figure S31. Seebeck coefficients of P3HT, T_{ref}, T19, T24 aligned thin films measured in the parallel (left) and perpendicular (right) direction to the rubbing direction.

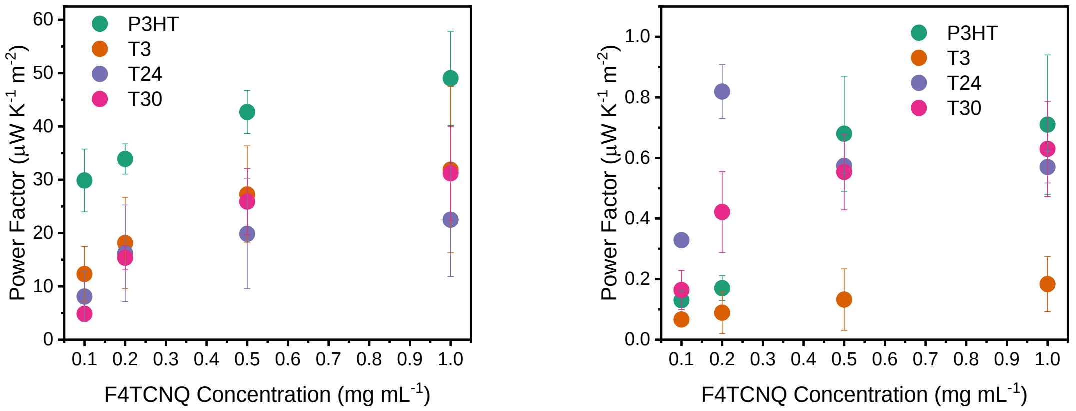

Figure S32. Calculated Power Factor results of P3HT, T_{ref}, T19, T24 aligned thin films in the parallel (left) and perpendicular (right) direction. The Power Factor was calculated using Power Factor = $S^2\sigma$ where S and σ are the Seebeck coefficient and conductivity respectively. The error bars correspond to the errors propagated from the conductivity measurements.

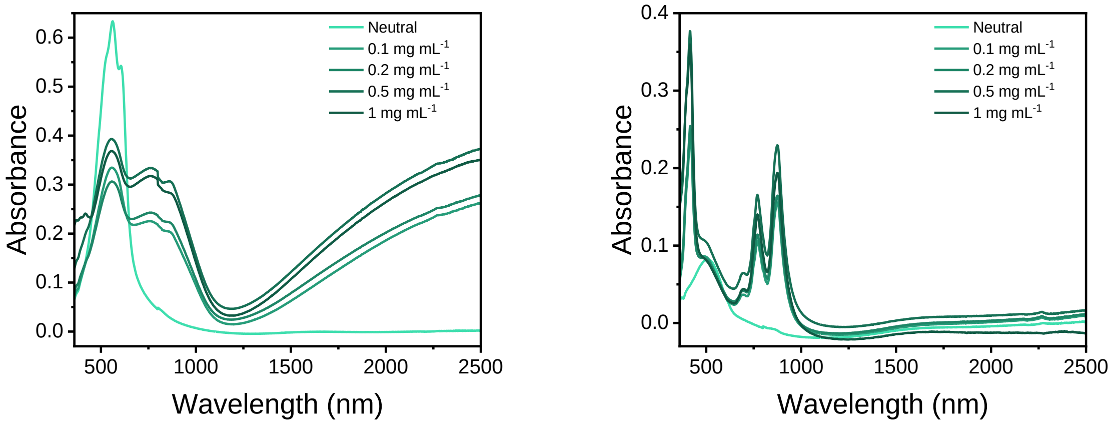

Figure S33. Polarised UV-Vis spectra of neutral and doped aligned P3HT thin films in the parallel (left) and perpendicular (right) direction to the rubbing direction.

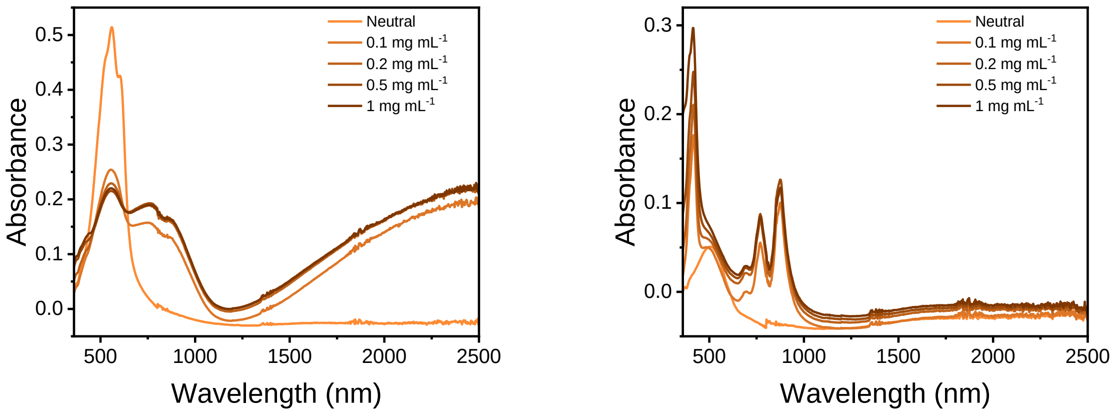

Figure S34. Polarised UV-Vis spectra of neutral and doped aligned T_{ref} thin films in the parallel (left) and perpendicular (right) direction to the rubbing direction.

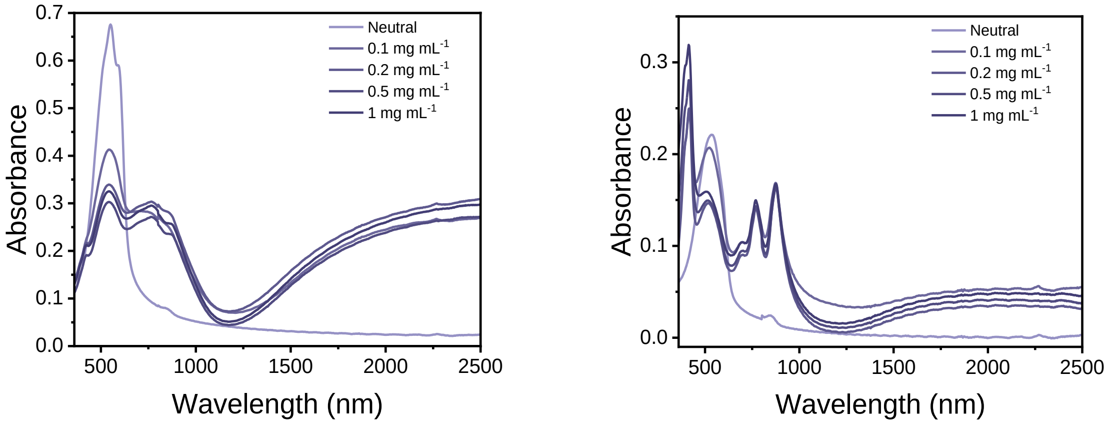

Figure S35. Polarised UV-Vis spectra of neutral and doped aligned T19 thin films in the parallel (left) and perpendicular (right) direction to the rubbing direction.

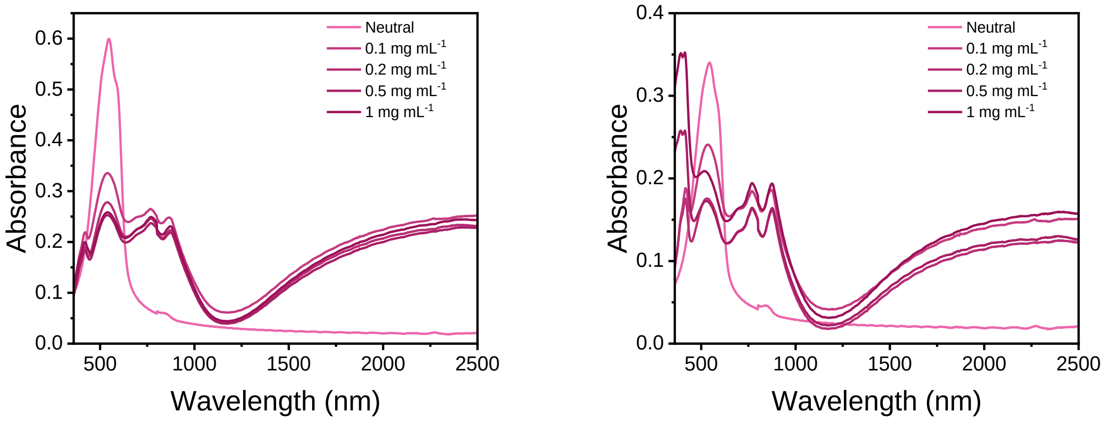

Figure S36. Polarised UV-Vis spectra of pristine and doped aligned T24 thin films in the parallel (left) and perpendicular (right) direction to the rubbing direction.

The polymer films were coated with a thin amorphous carbon film and subsequently floated on distilled water and recovered on TEM copper grids. TEM was performed in bright field and diffraction modes using a CM12 Philips microscope (120 kV) equipped with a MVIII (Soft Imaging System) camera. The 002 monomer periodicity reflection at 3.85 Å was used to calibrate the reticular distances in the ED patterns. The low dose system was used to avoid de-doping the polymer films under the electron beam (de-doping is evidenced by a change in reticular distances that recover values found for the pristine undoped P3HT films).

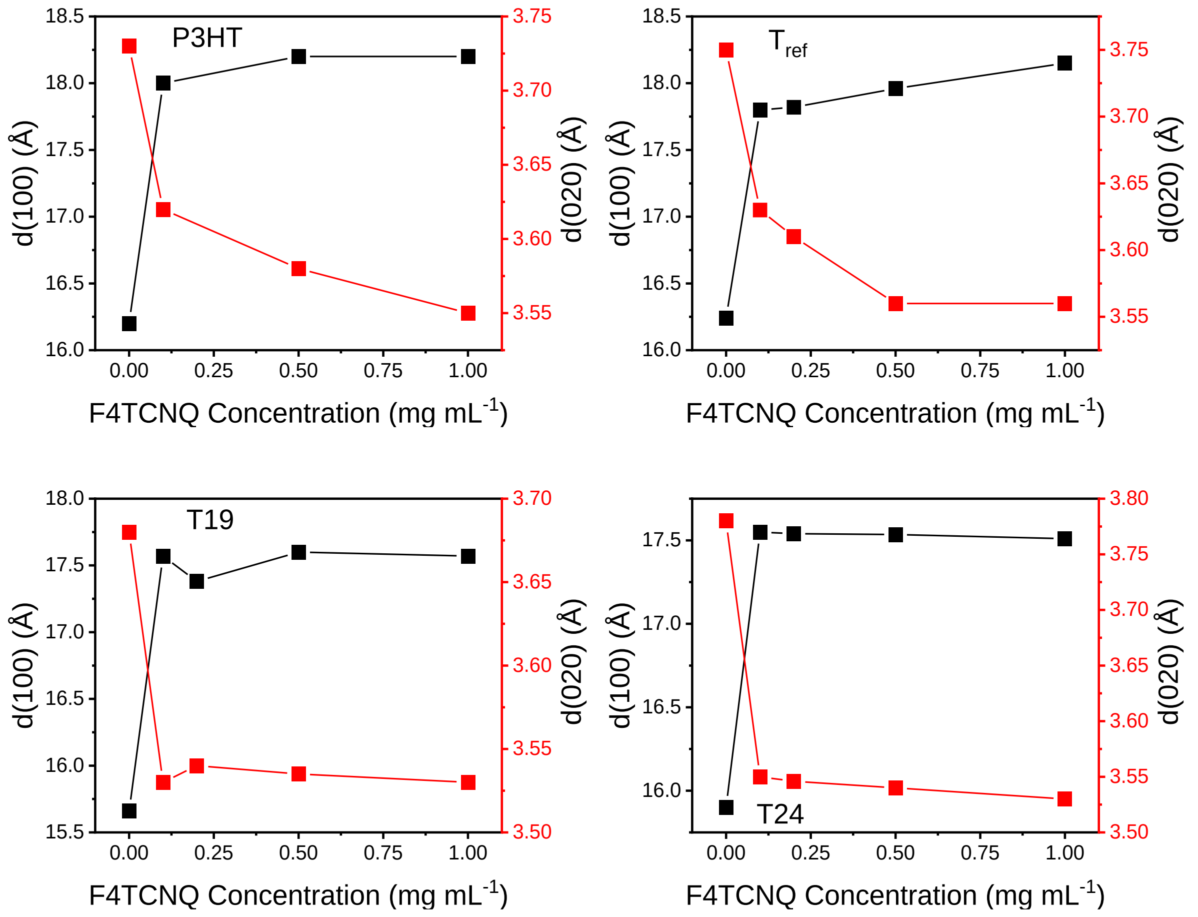

Figure S37. The d-spacings of the lamella (d(100)) and π-spacings (d(020)) of the aligned thin films doped from 0 to 1 mg mL^{-1} F4TCNQ solutions.

Table S9. Table showing the d-spacings of the (100) and (020) of doped aligned films at 1 mg ml^{-1}.

**Polymer**	Neutral		1 mg ml^{-1} Doped	
	d_{100} (Å)	d_{020} (Å)	d_{100} (Å)	d_{020} (Å)
**P3HT**	16.2	3.7	18.2	3.6
**T_{ref}**	16.2	3.8	18.2	3.6
**T19**	15.7	3.7	17.6	3.5
**T24**	15.9	3.8	17.5	3.5

To get a thickness for the aligned films a calibration curve of measured thickness vs absorption of the non-aligned films was created using AFM and UV-Vis spectroscopy. Thin films were fabricated as previously however solutions of 5, 10, 15 and 20 mg mL^{-1} were used to ensure the calibration curve fit in the region between 0 – 100 nm. A small section (~1 x 2 mm) of polymer thin films were measured using UV-Vis spectroscopy using a Shimadzu UV3600 UV-Vis-NIR spectrometer. Then a small section of the film was removed adjacent to the spot

measured using a wooden toothpick to ensure no scratching of the glass. Thickness measurements were taken from the removed section to the section where the absorption of the polymer film was measured, using the glass as the base line.

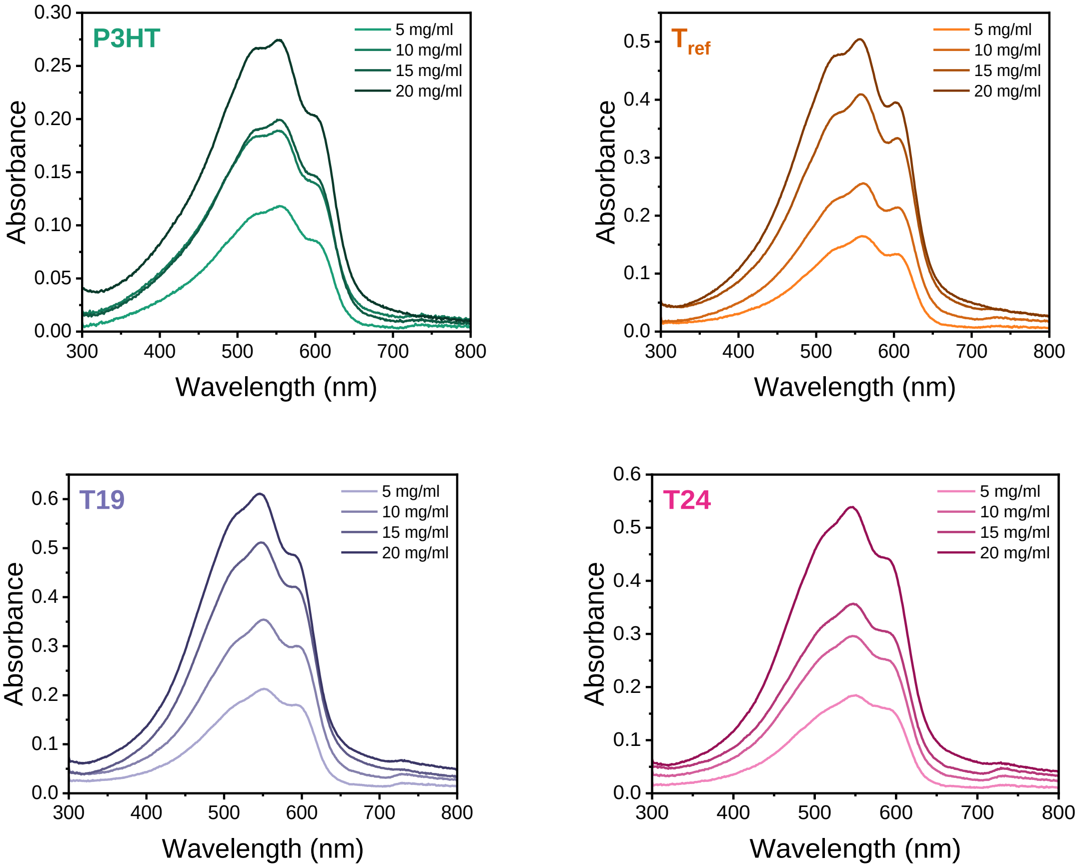

Figure S38. Thin film UV-Vis spectra of the polymer series of increasing thickness obtained by increasing the solution concentration in ODCB used for the calibration curves.

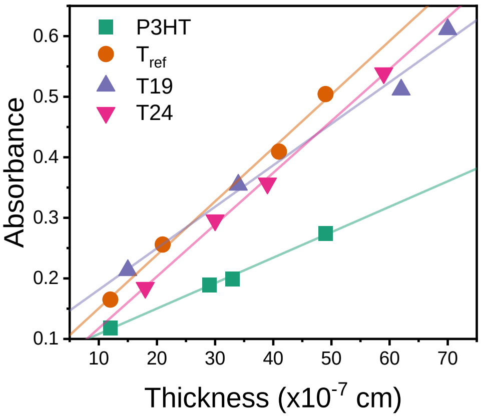

Figure S39. Calibration curves of thin films across the polymer series.

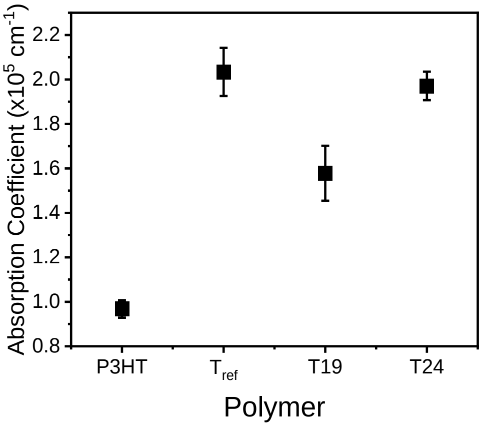

Figure S40. Plot of the optical absorption coefficient for each polymer. The absorption coefficient was calculated using the Beer-Lambert relation: absorption coefficient = 2.303(A/t), where A and t are the absorbance and film thickness (cm) respectively, and assuming that this latter is equivalent to the absorption path length. Error bars arise from the linear fit deviation of the calibration curve above.

15. References

(1) Finn, P. A.; Jacobs, I. E.; Armitage, J.; Wu, R. H.; Paulsen, B. D.; Freeley, M.; Palma, M.; Rivnay, J.; Sirringhaus, H.; Nielsen, C. B. Effect of polar side chains on neutral and p-doped polythiophene. *Journal of Materials Chemistry C* **2020**, *8* (45), 16216-16223. DOI: 10.1039/d0tc04290k.

(2) Qu, S.; Ming, C.; Yao, Q.; Lu, W.; Zeng, K.; Shi, W.; Shi, X.; Uher, C.; Chen, L. Understanding the Intrinsic Carrier Transport in Highly Oriented Poly(3-hexylthiophene): Effect of Side Chain Regioregularity. *Polymers (Basel)* **2018**, *10* (8). DOI: 10.3390/polym10080815 From NLM PubMed-not-MEDLINE.

(3) Biniek, L.; Pouget, S.; Djurado, D.; Gonthier, E.; Tremel, K.; Kayunkid, N.; Zaborova, E.; Crespo-Monteiro, N.; Boyron, O.; Leclerc, N.; et al. High-Temperature Rubbing: A Versatile Method to Align π-Conjugated Polymers without Alignment Substrate. *Macromolecules* **2014**, *47* (12), 3871-3879. DOI: 10.1021/ma500762x.

(4) Hamidi-Sakr, A.; Biniek, L.; Fall, S.; Brinkmann, M. Precise Control of Lamellar Thickness in Highly Oriented Regioregular Poly(3-Hexylthiophene) Thin Films Prepared by High-Temperature Rubbing: Correlations with Optical Properties and Charge Transport. *Advanced Functional Materials* **2016**, *26* (3), 408-420. DOI: 10.1002/adfm.201504096.

(5) Rivnay, J.; Mannsfeld, S. C.; Miller, C. E.; Salleo, A.; Toney, M. F. Quantitative determination of organic semiconductor microstructure from the molecular to device scale. *Chem Rev* **2012**, *112* (10), 5488-5519. DOI: 10.1021/cr3001109 From NLM PubMed-not-MEDLINE.

(6) Paterson, A. F.; Singh, S.; Fallon, K. J.; Hodsden, T.; Han, Y.; Schroeder, B. C.; Bronstein, H.; Heeney, M.; McCulloch, I.; Anthopoulos, T. D. Recent Progress in High-Mobility Organic Transistors: A Reality Check. *Advanced Materials* **2018**, *30* (36), 1801079. DOI: 10.1002/adma.201801079 From NLM Publisher.

(7) Freychet, G.; Huang, Y. X.; Tan, W. L.; Gilhooly-Finn, P. A.; Nielsen, C. B.; Sirringhaus, H.; McNeill, C. R. Drastic Enhancement of X-ray Scattering Contrast between Amorphous and Crystalline Phases of Poly(3-hexylthiophene) at the Sulfur K-Edge. *Acs Materials Letters* **2022**, *4* (5), 764-769. DOI: 10.1021/acsmaterialslett.2c00049.

(8) Hamidi-Sakr, A.; Biniek, L.; Bantignies, J. L.; Maurin, D.; Herrmann, L.; Leclerc, N.; Leveque, P.; Vijayakumar, V.; Zimmermann, N.; Brinkmann, M. A Versatile Method to Fabricate Highly In-Plane Aligned Conducting Polymer Films with Anisotropic Charge Transport and Thermoelectric Properties:

The Key Role of Alkyl Side Chain Layers on the Doping Mechanism. *Advanced Functional Materials* **2017**, *27* (25). DOI: 10.1002/adfm.201700173.
(9) Untilova, V.; Biskup, T.; Biniek, L.; Vijayakumar, V.; Brinkmann, M. Control of Chain Alignment and Crystallization Helps Enhance Charge Conductivities and Thermoelectric Power Factors in Sequentially Doped P3HT:F4TCNQ Films. *Macromolecules* **2020**, *53* (7), 2441-2453. DOI: 10.1021/acs.macromol.9b02389.
(10) Untilova, V.; Hynynen, J.; Hofmann, A. I.; Scheunemann, D.; Zhang, Y.; Barlow, S.; Kemerink, M.; Marder, S. R.; Biniek, L.; Muller, C.; et al. High Thermoelectric Power Factor of Poly(3-hexylthiophene) through In-Plane Alignment and Doping with a Molybdenum Dithiolene Complex. *Macromolecules* **2020**, *53* (15), 6314-6321. DOI: 10.1021/acs.macromol.0c01223 From NLM PubMed-not-MEDLINE.
(11) Vijayakumar, V.; Durand, P.; Zeng, H.; Untilova, V.; Herrmann, L.; Algayer, P.; Leclerc, N.; Brinkmann, M. Influence of dopant size and doping method on the structure and thermoelectric properties of PBTTT films doped with F6TCNNQ and F4TCNQ. *Journal of Materials Chemistry C* **2020**, *8* (46), 16470-16482. DOI: 10.1039/d0tc02828b.
